# Exposure of C57BL/6J mice to long photoperiod during early life stages increases body weight and alters plasma metabolomic profiles in adulthood

**DOI:** 10.14814/phy2.12974

**Published:** 2016-09-20

**Authors:** Tatsuhiro Uchiwa, Yusuke Takai, Ayako Tashiro, Mitsuhiro Furuse, Shinobu Yasuo

**Affiliations:** ^1^ Laboratory of Regulation in Metabolism and BehaviorFaculty of AgricultureKyushu UniversityFukuokaJapan

**Keywords:** Body weight, metabolome, perinatal period, photoperiod

## Abstract

Perinatal photoperiod is an important regulator of physiological phenotype in adulthood. In this study, we demonstrated that postnatal (0–4 weeks old) exposure of C57BL/6J mice to long photoperiod induced persistent increase in body weight until adulthood, compared with the mice maintained under short photoperiod. The expression of peroxisome proliferator‐activated receptor *δ*, a gene involved in fatty acid metabolism, was decreased in 10‐week‐old mice exposed to long photoperiod during 0–4 or 4–8 weeks of age. Plasma metabolomic profiles of adult mice exposed to a long photoperiod during the postnatal period (0–4 LD) were compared to those in the mice exposed to short photoperiod during the same period. Cluster analysis revealed that both carbon metabolic pathway and nucleic acid pathway were altered by the postnatal photoperiod. Levels of metabolites involved in glycolysis were significantly upregulated in 0–4 LD, suggesting that the mice in 0–4 LD use the glycolytic pathway for energy expenditure rather than the fatty acid oxidation pathway. In addition, the mice in 0–4 LD exhibited high levels of purine metabolites, which have a role in neuroprotection. In conclusion, postnatal exposure of C57BL/6J mice to long photoperiod induces increase in body weight and various changes in plasma metabolic profiles during adulthood.

## Introduction

Season of birth or perinatal photoperiod influences reproductive and somatic development in many mammalian species (Hoffmann 1978; Gorman 2001; Walton et al. 2011). The influences are mostly observed in developmental velocity or timing of maturation, for example, Djungarian hamsters raised from birth under short day condition (SD) delay puberty and growth, compared to those raised under long day condition (LD) (Hoffmann 1978). In addition, perinatal or gestational photoperiod has enduring effects on the physiological and behavioral phenotype in adulthood, including reproductive maturation and activity (van Haaster et al. 1993; Beery et al. 2008), immune response (Weil et al. 2006), and affective behaviors (Pyter and Nelson 2006). The enduring effects may be regulated by photoperiod‐induced imprinting of the neuronal activity in the brain; recent studies indicate that perinatal photoperiod affects brain physiology, including firing rate, responsiveness to noradrenergic stimulation, and intrinsic electrical properties in the serotonergic raphe nuclei (Green et al. 2015), and clock gene expression in the suprachiasmatic nucleus (Ciarleglio et al. 2011). Several effects appear to be mediated by a photoperiodic messenger melatonin (Dimitriadis et al. 2011) or other systems such as the hypothalamic‐pituitary‐adrenocortical axis (Toki et al. 2007).

In addition to photoperiod or season, a number of factors in the perinatal environment have great impact on offspring development, including maternal or postnatal nutrition, stress, and immune activity (Levin 2006). Among these, the most studied factors include the enduring effects of nutritional conditions in the early life stages on the metabolic characteristics in adulthood, such as weight gain, adiposity, and glucose homeostasis (Waterland and Garza 1999), which in turn is related to obesity and diabetes (Levin 2006). In animal models, maternal or early postnatal overnutrition alters functions, gene expression/metabolic profiles, or DNA methylation patterns in the hypothalamic neuropeptide circuits and leptin signal regulating energy balance (Davidowa and Plagemann 2000; Davidowa et al. 2003), lipid metabolism in the liver (Pereira et al. 2015), and insulin signaling in the skeletal muscle (Liu et al. 2013). Enduring effects of other environmental stimuli on these physiological parameters largely remain unclear.

For the investigation of photoperiod‐related physiology, high responsiveness to photoperiod is a mandatory property of animal models. Thus, most studies have used seasonal breeders such as Djungarian and Syrian hamsters, whereas nonseasonal breeders such as laboratory mice and rats have been considered inappropriate. However, we recently demonstrated high responsiveness of stress‐ and metabolism‐related properties to photoperiod in C57BL/6J mice. First, plasma corticosterone levels in C57BL/6J mice exhibited higher peaks under SD than those under LD (Otsuka et al. 2012). Second, the mice under SD exhibited high depression/anxiety‐like behaviors with low levels of the brain serotonin content (Otsuka et al. 2014; Kawai et al. 2015). Third, the mice under SD demonstrated higher body and epididymal fat weights, lower glucose tolerance, and higher plasma concentrations of a muscle degradation marker, than the mice under LD (Otsuka et al. 2014, 2015). Muscle fibers are comprised of slow‐twitch (I), fast‐twitch (IIb), and intermediate (IIa and IIx) types that have different properties in metabolism (Spangenburg and Booth 2003); the mice under SD exhibited higher percentage of IIb type than those under LD (Otsuka et al. 2015). Taken together, these results show that the C57BL/6J mouse is an appropriate model for use in photoperiodic regulation of stress and metabolism experiments.

The aim of this study was to clarify the enduring effects of photoperiod in the early life stages on body weight, fat and testicular weights, muscle fiber composition, expression of genes involved in fatty acid metabolism in muscle, peroxisome proliferator‐activated receptor (PPAR) *δ*,* γ*1, and *γ*2, and plasma metabolomics profiles in C57BL/6J mice to gain a comprehensive insight into the biological changes as a consequence of postnatal photoperiod. We also examined whether the enduring effects of photoperiod is dependent on the length of the light phase.

## Materials and Methods

### Animals

Male and female C57BL/6J mice (6 weeks old) were obtained from Japan SLC, Inc., and housed separately in plastic cages in groups of three or four. They were maintained under SD (6 h light: 18 h dark, 6L18D) for at least 3 weeks before mating in boxes in a room at 25 ± 1°C. Water and standard diet for laboratory rodents (MF, Oriental Yeast, Tokyo, Japan) were provided ad libitum. Thereafter, the mice were mated in a group of one‐two females and one male. Male offspring were used in the following experiments. From mating of males and females to weaning of offspring, the breeding diet (NMF, Oriental Yeast) was provided. All animal experiments in this study were conducted in accordance with the Guidelines for Animal Experiments in the Faculty of Agriculture, Kyushu University, as well as the law and a notification by the Japanese Government.

### Experiment 1

On the day of birth, the pups were separated into four groups (*n *=* *4–6, Table 1). Each group included the pups of 2–3 litters. The first group was maintained under SD from birth to 8 weeks of age (SDC). The second group was exposed to SD from birth to 4 weeks of age and LD (18L:6D) from 4 to 8 weeks of age (4–8 LD). The third group was exposed to LD from birth to 4 weeks of age and then SD from 4 to 8 weeks of age (0–4 LD). The fourth group was exposed to LD from birth to 8 weeks of age (0–8 LD). Mice from all groups were weaned at 3 weeks of age, and transferred to 12L:12D from 8 weeks of age. After weaning, body weights of all pups were measured once a week until 10 weeks of age. Mice were killed 2 h after lights‐on under isoflurane anesthesia at 10 weeks of age. Epididymal fat and testes were dissected and weighed. Trunk blood samples were collected in heparinized tubes and centrifuged at 3000 × *g* for 10 min at 4°C. Plasma was collected and stored at −80°C. The gastrocnemius muscle was dissected out, quickly frozen in liquid nitrogen, and stored at −80°C. The muscle samples were homogenized in a mortar chilled in liquid nitrogen, and separated into two portions for real‐time polymerase chain reaction (PCR) and muscle fiber type analysis.

**Table 1:** Experimental schedule in this study

Experiment	Group^a^	0–4 weeks^b^	4–8 weeks^b^	8–10 weeks
1	SDC	SD	SD	12L:12D
1	0–4 LD	LD	SD	12L:12D
1	4–8 LD	SD	LD	12L:12D
1	0–8 LD	LD	LD	12L:12D
2	6L:18D	6L:18D	6L:18D	12L:12D
2	16L:8D	16L:8D	16:L8D	12L:12D
2	18L:6D	18L:6D	18:L6D	12L:12D
2	20L:4D	20L:4D	20:L4D	12L:12D
2	22L:2D	22L:2D	22:L2D	12L:12D

a Dams in all groups were maintained under 6L:18D during gestational period.

b SD: 6L:18D, LD: 18L:6D.

### Experiment 2

On the day of birth, the pups were separated into five groups (*n *=* *4–12, Table 1). Each group included the pups of 2–3 litters. The photoperiod from birth to 8 weeks of age was set as 6L:18D, 16L:8D, 18L:6D, 20L:4D, and 22L:2D. They were weaned at 3 weeks of age and transferred to 12L:12D from 8 weeks of age. Measurements of body weight and epididymal fat and testicular weights were performed as described in Experiment 1.

### Composition of muscle fiber types

The muscle fiber types in the gastrocnemius muscle were analyzed using sodium dodecyl sulfate (SDS)‐polyacrylamide gel electrophoresis of myosin heavy‐chain isoforms, IIa, IIb, and IIx, according to a previous report (Mizunoya et al. 2008). Muscle samples were homogenized in a solution containing 10% w/v SDS, 40 mmol/L dithiothreitol, 5 mmol/L EDTA, and 0.1 mol/L Tris‐HCl buffer. The separating gel consisted of 35% v/v glycerol, 8% w/v acrylamide‐*N,N′*‐methylenebisacrylamide (99:1), 0.2 mol/L Tris‐HCl (pH 8.8), 0.1 mol/L glycine, 0.4% w/v SDS, 0.1% w/v ammonium persulfate, and 0.05% v/v *N,N,N′,N′*‐tetramethylethylenediamine. Samples containing 100 ng protein were applied to the gel and electrophoresed at a constant voltage of 140 V for 23 h except for the first 40 min, where the maximum current was limited to 10 mA. The gel unit remained at 4°C throughout the electrophoresis. After the run, gels were stained using a silver stain kit (Silver Stain KANTO III; Kanto Chemicals, Tokyo, Japan) and a picture of the bands was taken by a digital imager (Fusion SL4, Vilber Lourmat, Marne‐la‐Vallee Cedex, France). We could not clearly separate the bands of IIa and IIx, so the bands of IIb and IIa + IIx were quantified densitometrically using ImageJ 1.47 software (National Institute of Health, Bethesda, MD). The data are expressed as percentage of IIb content (fast‐twitch fiber) to the sum of IIa, IIb, and IIx contents.

### Real‐time PCR

Total RNA was extracted from the muscle samples using RNAiso Plus (Takara; Shiga, Japan) according to the manufacturer's protocol. cDNA was synthesized using 1 μg of total RNA and PrimeScript RT reagent Kit with gDNA Eraser (Takara) according to the manufacturer's protocol. Real‐time PCR was performed using Stratagene Mx3000P (Agilent Technologies; Santa Clara, CA) with a denaturation step at 95°C for 30 sec, then 40 cycles of PCR amplification at 95°C for 5 sec, and at 58°C for 30 sec. Primer sequences of PPAR*δ*,* γ*1, and *γ*2 are listed in Table 2. Each mRNA level was calculated using threshold cycles for the amplification of unknown samples compared with those of four concentrations of standards. The calculated levels were normalized using mRNA levels of hypoxanthine phosphoribosyltransferase (HPRT). Melting curve analysis was performed for each gene to confirm the specificity of PCR conditions.

**Table 2:** Primer sequences used in this study

Gene	Sequence (5′–3′)	Melting temp
PPAR*δ*		
Forward	GCAGCCTCAACATGGAATGTC	66.8
Reverse	GAGCTTCATGCGGATTGTCC	66.9
PPAR*γ1*		
Forward	TTTTCCGAAGAACCATCCGATT	67.3
Reverse	ATGGCATTGTGAGACATCCCC	68.1
PPAR*γ2*		
Forward	TCGCTGATGCACTGCCTATG	67.4
Reverse	GAGAGGTCCACAGAGCTGATT	62.4
HPRT		
Forward	ATACAGGCCAGACTTTGTTGGATT	66.5
Reverse	TCACTAATGACACAAACGTGATTCAA	66.7

### Metabolome analysis

Three animals each were chosen from the SDC and 0–4 LD groups in Experiment 1. Their plasma samples were used for metabolome analysis (Human Metabolome Technologies Inc.; Tsuruoka, Japan). Plasma samples (50 μL) were transferred into 450 μL of methanol containing 10 μmol/L of internal standard. Then, 500 μL of chloroform and 200 μL of water were added and mixed, and the samples were centrifuged at 2300 × *g* for 5 min at 4°C. The water phase was collected and filtered through a filter unit (Ultrafree‐MC PLHCC, 5 kDa, Millipore; Billerica, MA) by centrifugation at 9100 × *g* for 120 min at 4°C. The filtrates were dried and dissolved in 25 μL of water and applied to capillary electrophoresis time‐of‐flight mass spectrometry (CE‐TOFMS) using an Agilent CE‐TOFMS system (Agilent Technologies) with a fused silica capillary, internal diameter 50 μm × 80 cm. The detected peaks were processed using automatic integration software (MasterHands ver. 2.16.0.15, Keio University; Tokyo, Japan), and aligned according to their m/z values and normalized migration times. Peak areas were normalized against those of internal standards. The relative area values were further normalized by the amount of samples. The detected peaks were annotated using m/z values and normalized migration times with the databases of Human Metabolome Technologies.

### Statistical analysis

Body weights during weeks 3–10 in Experiments 1 and 2 were analyzed by repeated two‐way analysis of variance (ANOVA) with group and age as factors. In Experiment 1, body weight, epididymal fat, and testicular weight, as well as composition of muscle fiber types and muscle genes expression at 10 weeks of age were analyzed by two‐way ANOVA with photoperiod during 0–4 and 4–8 weeks of age as factors, followed by a Bonferroni multiple comparison test. In Experiment 2, body weight, epididymal fat, and testicular weight at 10 weeks of age were analyzed by one‐way ANOVA followed by a Bonferroni multiple comparison test.

Metabolome data were analyzed using principal component analysis (PCA, SampleStat ver. 3.14, Human Metabolome Technologies) and hierarchical cluster analysis (HCA, PeakStat ver. 3.18, Human Metabolome Technologies). Heat maps were generated by coloring the values of all data according to the color scale. Relative area values were used for calculation of the ratio of 0–4 LD to SDC groups and for comparison between 0–4 LD and SDC groups using Student's *t*‐test. Values were considered significantly different at *P *<* *0.05.

## Results

### Effect of postnatal photoperiod on body weight, epididymal fat, and testicular weights

**Figure 1:**
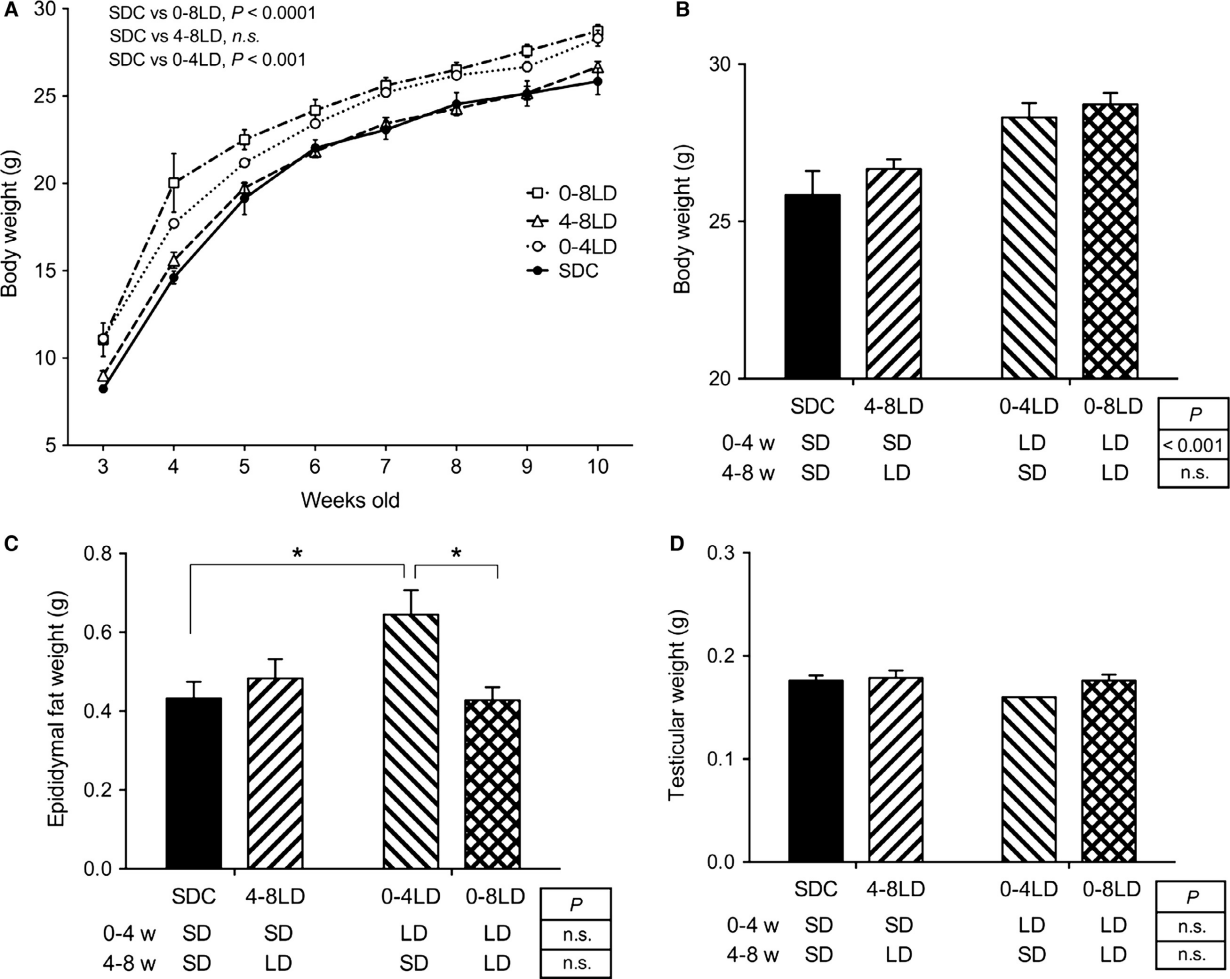
Effect of postnatal photoperiod on body weight, epididymal fat, and testicular weights in C57BL/6J mice. (A) Temporal changes in body weight. Body weights in mice in 0–4 LD and 0–8 LD were significantly higher than mice in SDC. *P*‐values, Bonferroni multiple comparison test following two‐way ANOVA. (B–D) Body weight (B), epididymal fat weight (C), and testicular weight (D) in the mice at 10 weeks of age. *P*‐values, main effects of photoperiod during 0–4 and 4–8 weeks of age, detected by two‐way ANOVA. *P <* *0.05, Bonferroni multiple comparison test. Data are presented as means ± SEM.

In Experiment 1, body weights of mice during 3–10 weeks of age in 0–4 LD and 0–8 LD, but not in 4–8 LD, were significantly higher than those in SDC (0–4 LD: *P *<* *0.001, 0–8 LD: *P *<* *0.0001) with no significant interaction with age (Fig. 1A). Comparison of body weight at 10 weeks of age using two‐way ANOVA showed that photoperiod during 0–4 weeks of age, but not 4–8 weeks of age, had a significant effect (*P *<* *0.001), without any significant interaction between them (Fig. 1B). Exposure of animals to LD during 0–4 weeks of age exhibited significantly higher body weight compared to those exposed to SD, irrespective of photoperiod during 4–8 weeks of age (Fig. 1B). On the other hand, a significant interaction between photoperiod during 0–4 and 4–8 weeks of age was detected in epididymal fat weight (*P *<* *0.05). Post hoc analysis clarified that epididymal fat weight in the 0–4 LD group was significantly higher than that in the SDC and 0–8 LD groups (*P *<* *0.05) (Fig. 1C). Testicular weight was not different among groups (Fig. 1D).

**Figure 2:**
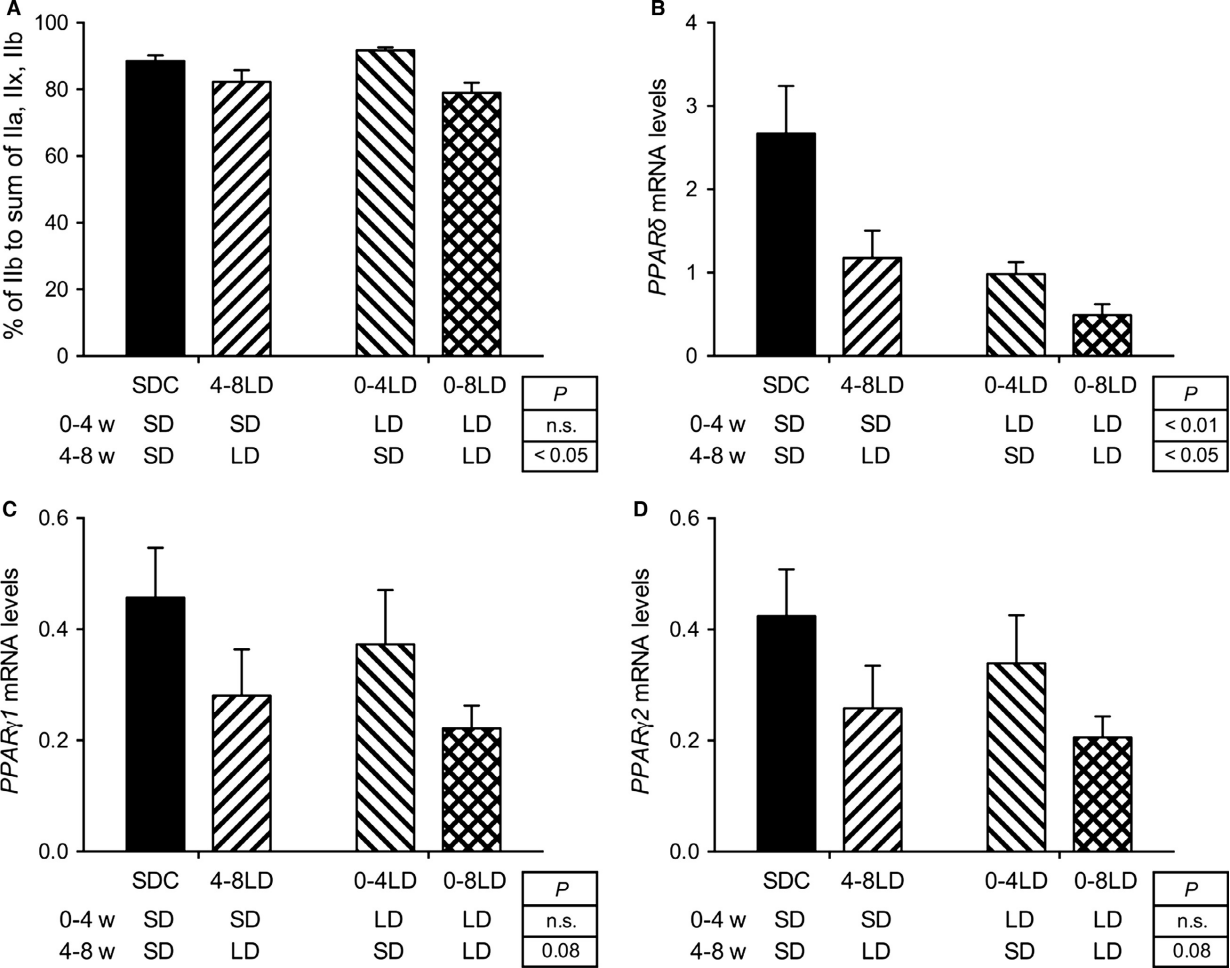
Effect of postnatal photoperiod on composition of muscle fiber type and gene expression in C57BL/6J mice. (A) Composition of muscle fiber type. Percentage of IIb type was significantly suppressed by exposure of mice to long photoperiod during 4–8 weeks of age. (B–D) Expression of PPAR *δ* (B), *γ*1 (C), and *γ*2 (D) in the mice at 10 weeks of age. PPAR *δ* expression was significantly suppressed by exposure of mice to long photoperiod during 0–4 or 4–8 weeks of age. *P*‐values, main effects of photoperiod during 0–4 and 4–8 weeks of age, detected by two‐way ANOVA. Data are presented as means ± SEM.

Percentage of type IIb of myosin heavy chain isoforms in the gastrocnemius muscle was significantly affected by photoperiod during 4–8 weeks of age, but not during 0–4 weeks of age, without any significant interaction between them (*P *<* *0.05) (Fig. 2A). It was lower in the mice exposed to LD from 4 to 8 weeks of age than in those exposed to SD during the same period (Fig. 2A). The expression of PPAR*δ* was significantly lowered by exposure of mice to LD during 0–4 (*P *<* *0.01) or 4–8 (*P *<* *0.05) weeks of age (Fig. 2B). The expression of PPAR*γ*1 and *γ*2 was tended to be lower in mice exposed to LD during 4–8 weeks of age (*P *=* *0.08) (Fig. 2C and D).

### Effect of day length during postnatal period on body weight, epididymal fat, and testicular weights

**Figure 3:**
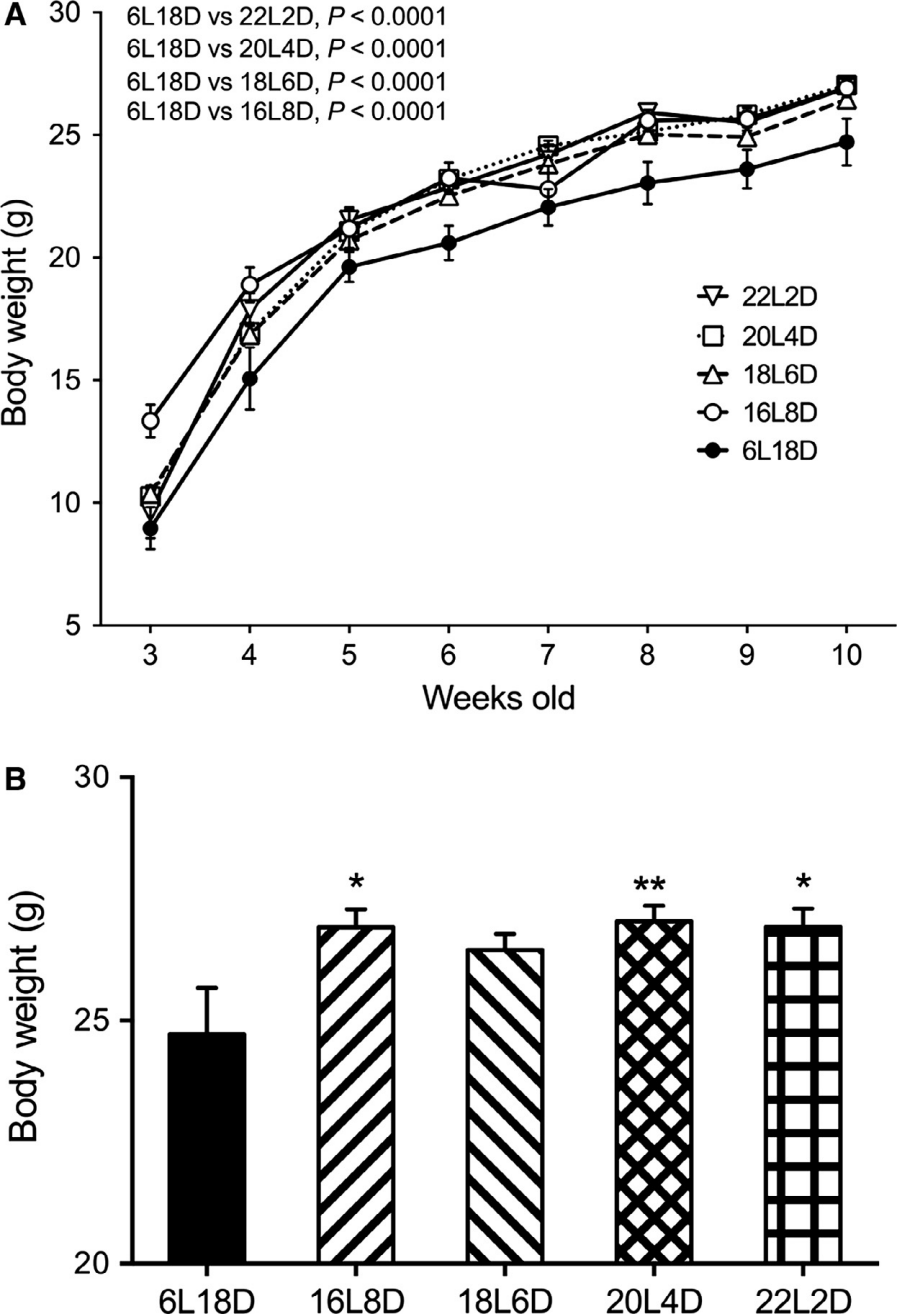
Effect of day length during postnatal period on body weight. (A) Temporal changes in body weight. The mice exposed to long photoperiod for more than 16 h exhibited significant increases of body weight. *P*‐values, Bonferroni multiple comparison test following two‐way ANOVA. (B) Body weight at 10 weeks of age. *P <* *0.05, **P <* *0.01, Bonferroni multiple comparison test. Data are presented as means ± SEM.

In Experiment 2, body weights of mice during 3–10 weeks of age in 16L:8D, 18L:6D, 20L:4D, and 22L:2D groups were significantly higher than those in 6L:18D (*P *<* *0.0001) with no significant interaction with age (Fig. 3A). There was no apparent difference among the 16L:8D, 18L:6D, 20L:4D, and 22L:2D groups. Body weights of mice at 10 weeks of age in 16L:8D, 20L:4D, and 22L:2D groups were significantly higher than those in the 6L:18D group (16D:8D and 22L:2D, *P *<* *0.05; 18L:6D, *P *<* *0.01) (Fig. 3B). Epididymal fat and testicular weights were not significantly different among groups.

### Metabolomic profiles in mice exposed to LD postnatally

**Figure 4:**
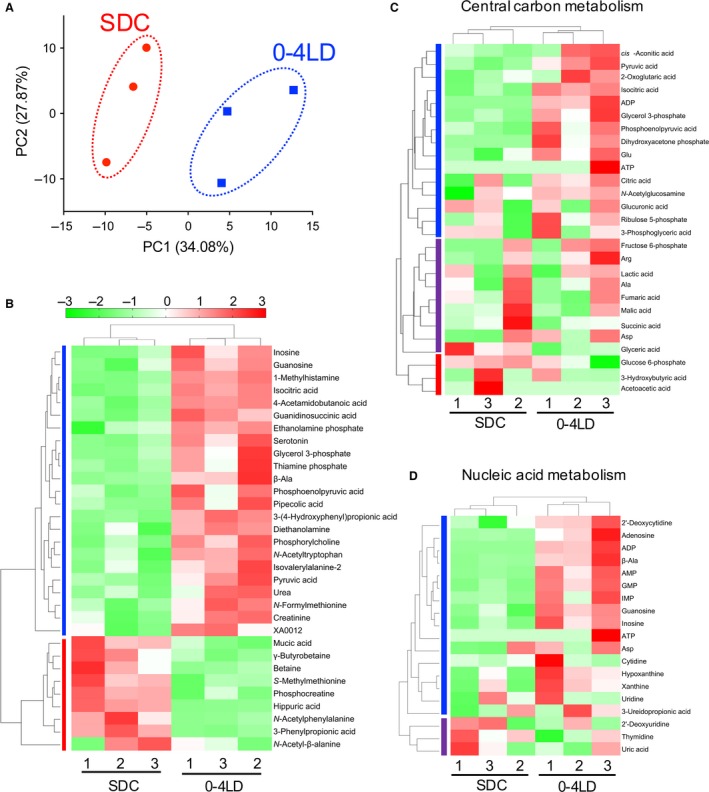
Effect of postnatal photoperiod on plasma metabolomics profiles. (A) In the principle component analysis, the first principle component (PC1) clearly distinguished 0–4 LD and SDC. (B–D) Heat map and result of hierarchical cluster analysis in the metabolites with significant differences between 0–4 LD and SDC (B) or metabolites involved in central carbon metabolism (C) and nucleic acid metabolism (D).

Metabolomic analysis was conducted in the plasma of 0–4 LD and SDC mice at 10 weeks of age in Experiment 1. In total, 219 metabolites (124 cations and 95 anions) were detected (Table S1). PCA revealed that the first principal component (PC1) efficiently separated the groups (Fig. 4A). Among the 219 metabolites detected, levels of 32 metabolites were significantly different between 0–4 LD and SDC (Fig. 4B, Table 3). HCA and heat map drawing using these 32 metabolites revealed that SDC and 0–4 LD samples were clearly clustered (Fig. 4B). These metabolites were classified into two big clusters, metabolites with 0–4 LD > SDC (23 metabolites) and those with 0–4 LD < SDC (9 metabolites). Each cluster included metabolites in several metabolic pathways (Table 3). Thus, we further performed HCA for levels of detected metabolites involved in each pathway (central carbon metabolism, 27 metabolites; fatty acids and amino acid metabolism, 33 metabolites; urea cycle and related amino acid metabolism, 37 metabolites; branched‐chain and aromatic amino acids metabolism, 18 metabolites; and nucleic acid metabolism, 19 metabolites). Among them, metabolites involved in central carbon metabolism and nucleic acid metabolism were clearly clustered into SDC and 0–4 LD (Fig. 4C and D).

**Table 3:** Plasma metabolites that changed with postnatal photoperiod

Compound name	Ratio^a^	*P*‐value	HMT pathway
Inosine	6.8	0.0133	Nucleic acid metabolism
Guanosine	2.9	0.0342	Nucleic acid metabolism
1‐Methylhistamine	3.3	0.0002	Urea cycle and related amino acid metabolism
Isocitric acid	1.2	0.0009	Central carbon metabolism
4‐Acetamidobutanoic acid	1.9	0.0003	
Guanidinosuccinic acid	1.4	0.0031	
Ethanolamine phosphate	1.6	0.0171	
Serotonin	5.4	0.0099	Branched‐chain and aromatic amino acids metabolism
Glycerol 3‐phosphate	1.5	0.0333	Central carbon metabolism/Fatty acid and amino acid metabolism
Thiamine phosphate	1.4	0.0396	Coenzyme metabolism
*β*‐Ala	1.7	0.0187	Urea cycle and related amino acid metabolism/Nucleic acid metabolism/Coenzyme metabolism
Phosphoenolpyruvic acid	1.9	0.0352	Central carbon metabolism
Pipecolic acid	1.2	0.0301	Fatty acid and amino acid metabolism
3‐(4‐Hydroxyphenyl)propionic acid	4.0	0.0006	
Diethanolamine	1.4	0.0169	
Phosphorylcholine	1.7	0.0080	Fatty acid and amino acid metabolism
*N*‐Acetyltryptophan	1.3	0.0086	
Isovalerylalanine‐2 N‐Acetylleucine‐2	1.6	0.0340	
Pyruvic acid	1.7	0.0157	Central carbon metabolism
Urea	1.1	0.0427	Urea cycle and related amino acid metabolism
*N*‐Formylmethionine	1.6	0.0174	
Creatinine	1.1	0.0281	Urea cycle and related amino acid metabolism
XA0012	1.2	0.0484	
Mucic acid	0.8	0.0146	
*γ*‐Butyrobetaine	0.8	0.0261	Fatty acid and amino acid metabolism
Betaine	0.7	0.0386	Fatty acid and amino acid metabolism
*S*‐Methylmethionine	0.7	0.0129	
Phosphocreatine	0.3	0.0080	Urea cycle and related amino acid metabolism
Hippuric acid	0.08	0.0005	
*N*‐Acetylphenylalanine	0.4	0.0184	
3‐Phenylpropionic acid	0.3	0.0016	
*N*‐Acetyl‐*β*‐alanine	0.4	0.0352	

a Ratio of 0–4 LD to SDC.

**Figure 5:**
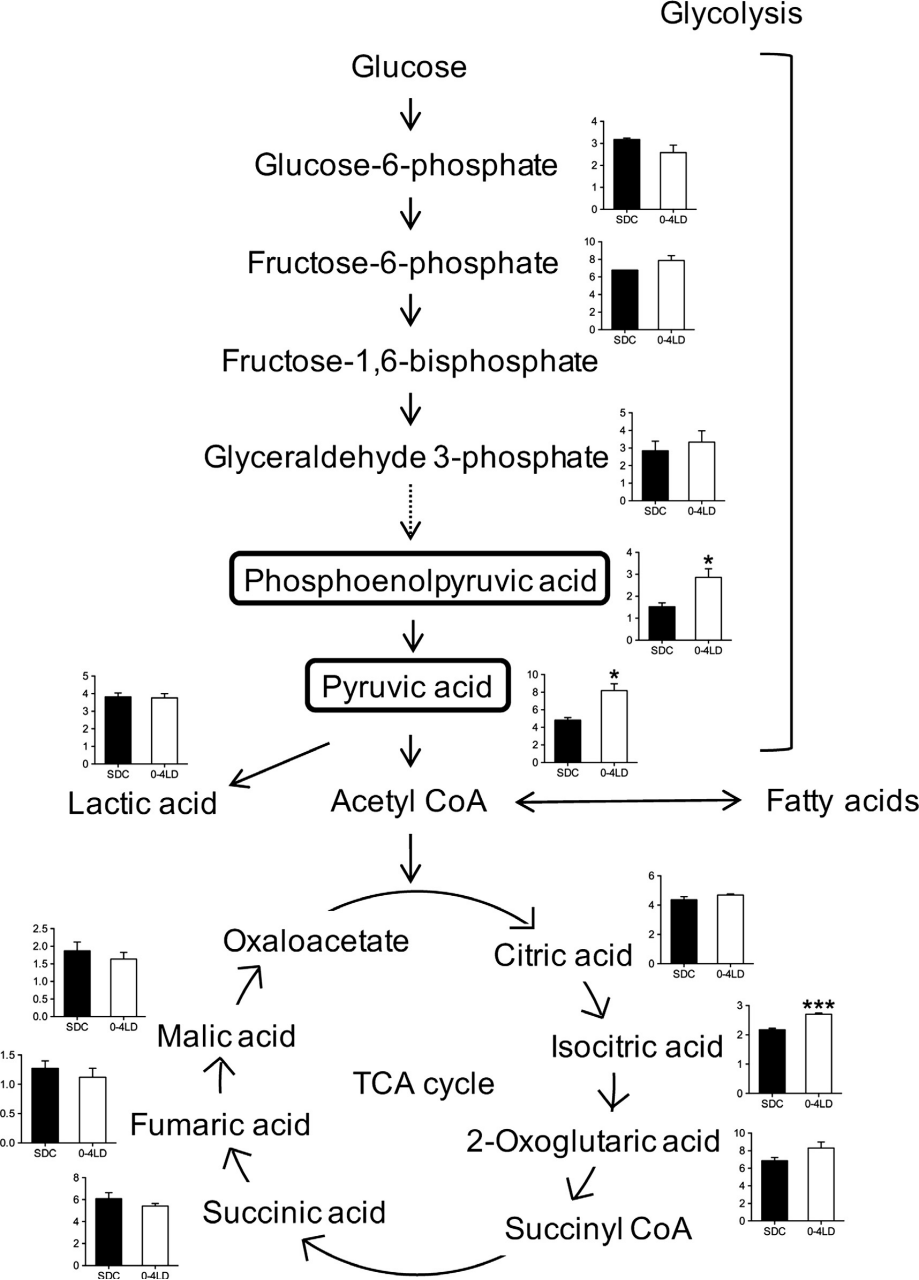
Carbon metabolic pathway. Graphs for each metabolite show the relative area values of metabolites in SDC and 0–4 LD. Levels of phosphoenolpyruvic acid and pyruvic acid were significantly higher in 0–4 LD than in SDC. *P <* *0.05, ***P <* *0.001, *t*‐test.

Metabolites in central carbon metabolism were clustered into three big clusters of metabolites (Fig. 4C). The first cluster involved metabolites in the glycolytic pathway, that is, pyruvic acid and phosphoenolpyruvic acid, which were significantly higher in 0–4 LD than SDC (Fig. 5). This cluster also involved isocitric acid, a metabolite in the tricarboxylic acid (TCA) cycle. The second cluster involved various metabolites in the downstream pathway of pyruvic acid: lactic acid and several metabolites in the TCA cycle, that is, fumaric acid, malic acid, and succinic acid, which were not significantly different between 0–4 LD and SDC (Fig. 5). The third cluster involved glucose 6‐phosphate and ketone bodies, that is, 3‐hydroxybutyric acid and acetoacetic acid, which were not significantly different between 0–4 LD and SDC.

**Figure 6:**
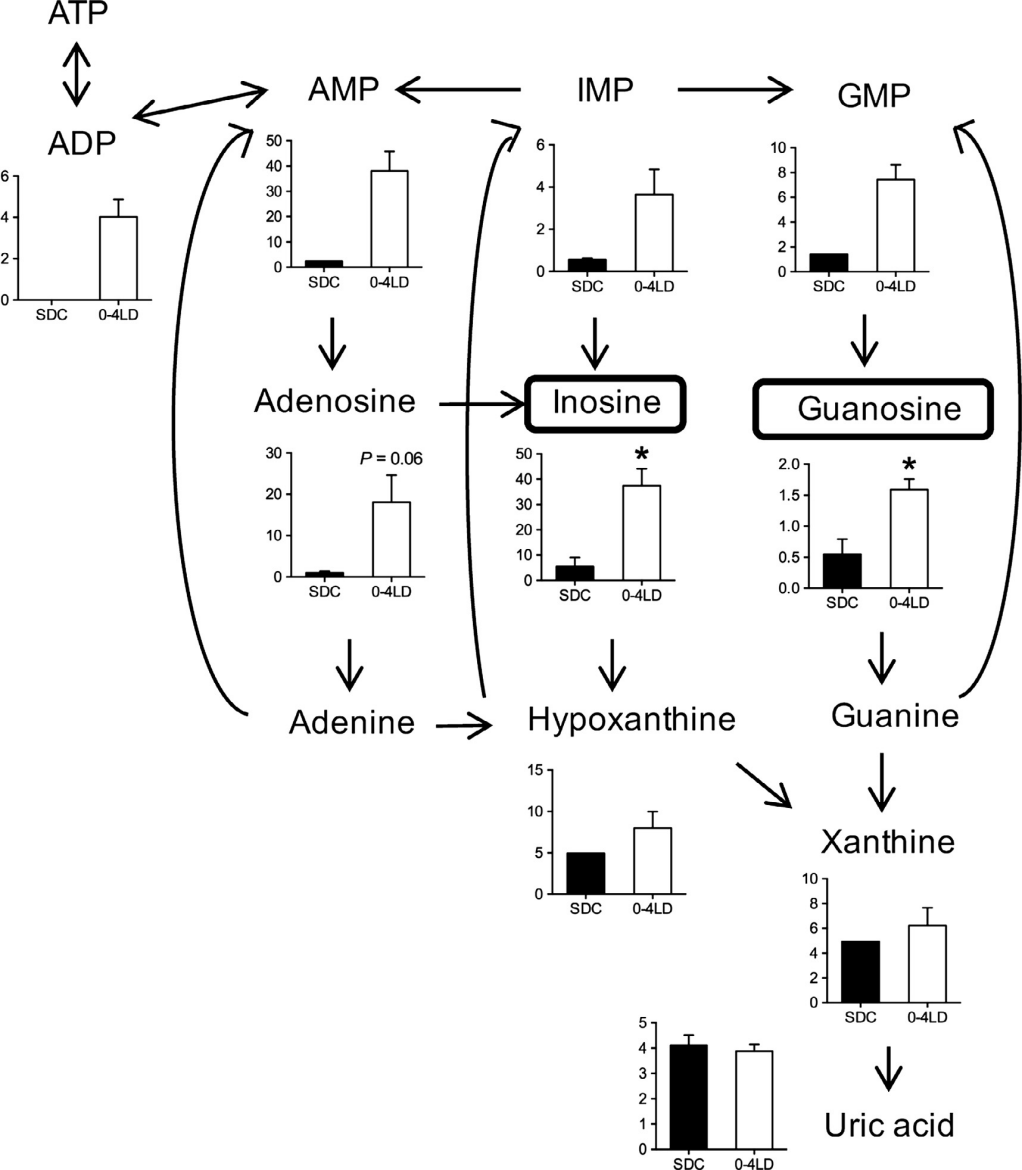
Purine metabolic pathway. Graphs for each metabolite show the relative area values of metabolites in SDC and 0–4 LD. Levels of inosine and guanosine were significantly higher in 0–4 LD than in SDC. *P <* *0.05, *t‐*test.

Metabolites in nucleic acid metabolism were clustered into two clusters (Fig. 4D). The first cluster involved various metabolites in the purine metabolic pathway. In addition, profound effects were observed in the levels of inosine (6.8‐fold in 0–4 LD compared to SDC) and guanosine (2.9‐fold) (Table 3). Thus, we inspected the metabolites in purine metabolism (Fig. 6). Adenosine levels tended to be higher in 0–4 LD than in SDC (*P *=* *0.06). Adenosine diphosphate (ADP), adenosine monophosphate (AMP), inosine monophosphate (IMP), and guanosine monophosphate (GMP) were constantly detected in the 0–4 LD samples, but their levels were low or not detected in the SDC samples. On the other hand, concentrations of hypoxanthine, xanthine, and uric acid were not significantly different between 0–4 LD and SDC.

Several metabolites related to creatine metabolism and kidney function were altered by postnatal photoperiod. Phosphocreatine levels were lower in 0–4 LD than in SDC, whereas levels of urea and creatinine were higher in 0–4 LD than in SDC (Table 3). Other metabolites that showed profound changes were serotonin (5.4‐fold in 0–4 LD compared to SDC), 3‐(4‐hydroxyphenyl) propionic acid (4.0‐fold), 1‐methylhistamine (3.3‐fold), and hippuric acid (0.08‐fold) (Table 3). With regard to serotonin and 1‐methylhistamine, upstream metabolites (tryptophan and histidine, respectively) or downstream metabolites (5‐methoxyindoleacetic acid and 1‐methyl‐4‐imidazoleacetic acid, respectively) were not significantly different between 0–4 LD and SDC.

## Discussion

This study clearly showed that postnatal (0–4 weeks old) exposure of C57BL/6J mice to LD resulted in higher body weight until adulthood, compared to those maintained under SD. This result is consistent with a previous study which demonstrated that body weight in Djungarian hamsters raised from birth under 16L:8D photoperiod increased steadily compared to those raised under 8L:16D (Hoffmann 1978). Our study further showed that after 4 weeks of age, photoperiod did not affect body weight, but the effect of postnatal photoperiod persisted until at least 10 weeks of age. The enduring effects of photoperiod are known in reproductive functions of hamsters (van Haaster et al. 1993; Beery et al. 2008). However, this study showed no effect of postnatal photoperiod on testicular development in C57BL/6J mice, probably due to the lack of detectable levels of melatonin (Ebihara et al. 1986) that transfers perinatal photoperiodic information to gonadal development (Tuthill et al. 2005). Furthermore, epididymal fat weight did not mirror body weight. These results suggest that postnatal LD did not simply promote the growth of mice, but it specifically programmed the energy set point that determines body weight and metabolism during adulthood.

In our previous study, adult C57BL/6J mice exposed to SD for 3 weeks exhibited higher body weight and epididymal fat weight than the mice under LD (Otsuka et al. 2015). This is inconsistent with this study which showed that after 4 weeks of age, photoperiod did not affect body weight. The discrepancy may be explained by the timing of photoperiod shift from SD to LD or LD to SD, that is, 4 weeks of age in this study and adult in the previous study. Sensitivity to photoperiod shift may be established in adults and the mechanism underlying photoperiodic response of body weight differ between young and adult mice. In line with this hypothesis, Syrian hamsters exposed to SD show increased body weight only when they become adults (Bartness and Wade 1985).

The effect of postnatal photoperiod on body weight was not correlated with absolute length of day, but photoperiod of more than 16 h day length elicited an increase in body weight. These data suggest that the changes in body weight were not a consequence of a shorter night with low activity levels or food intake. Although we did not measure activity levels and food intake in this study, our previous studies showed no major changes in wheel‐running activity, core body temperature, or food intake between mice maintained under LD and SD, even though night length was 8 and 16 h, respectively (Otsuka et al. 2014; Kawai et al. 2015).

Effect of postnatal (0–4 weeks old) photoperiod was not observed in the composition of muscle fiber type, despite clear changes in body weight. Rather, they were sensitive to photoperiod after 4 weeks of age. Considering that perinatal muscle growth with increased number of myofiber and myonuclei occurred until postnatal day 21 in mice (White et al. 2010), sensitivity of muscle fiber type to photoperiod may need developed muscle fibers. Nevertheless, expression of PPAR*δ* was significantly inhibited by exposure of mice to LD during 0–4 and 4–8 weeks of age. PPARs are nuclear receptors that are activated by fatty acids and their derivatives, and have multiple functions in metabolism including fatty acid oxidation and translocation, energy balance, and lipid metabolism (Ehrenborg and Krook 2009). The expression of PPAR*δ* is not only upregulated by exercise or fasting, in which fatty acids are needed as an energy source (Pereira et al. 2015), but are also regulated by nutritional condition during pre‐ and early‐postnatal periods (Hou et al. 2013; Chang et al. 2016). Lower expression of PPAR*δ* in the mice exposed to LD postnatally suggests that these animals mainly use a glycolytic pathway for energy expenditure rather than a fatty acid oxidation pathway. This interpretation is supported by high levels of plasma metabolites involved in the glycolytic pathway in 0–4 LD mice, as described below. It should be noted that muscle fiber type IIb has high glycolytic capacity (Spangenburg and Booth 2003), but muscle fiber type composition was not altered by postnatal photoperiod. Thus, the regulation of PPAR*δ* expression appears to be independent of muscle fiber regulation.

In the metabolomic analysis of plasma, levels of metabolites involved in carbon metabolism exhibited global alteration between 0–4 LD and SDC mice, with significant upregulation of metabolites in the glycolytic pathway (phosphoenolpyruvic acid and pyruvic acid) and TCA cycle (isocitric acid) in 0–4 LD mice. However, levels of lactic acid and several metabolites in the TCA cycle were not affected by the postnatal photoperiod. These results suggest that the glycolytic pathway and a part of the TCA cycle were activated in 0–4 LD. As the TCA cycle occurs in the matrix of mitochondria, tissue levels of metabolites in the TCA cycle may show distinct upregulation in 0–4 LD mice. In the enzymatic activity levels, high levels of pyruvic acid suggest that the enzymatic activity of pyruvate dehydrogenase complex that metabolizes pyruvic acid to acetyl CoA may be suppressed in 0–4 LD mice. Because the rate of glycolysis is activated and that of fatty acid oxidation, which is regulated by PPAR*δ*, is decreased by insulin in skeletal muscle (Dimitriadis et al. 2011), insulin action may be promoted in 0–4 LD mice. This hypothesis is supported by the result that the plasma of 0–4 LD mice contained high levels of serotonin, which improves insulin resistance in high fat diet‐induced obesity in mice (Watanabe et al. 2016). However, activated glycolysis cannot explain increased body weight in 0–4 LD mice, because enhancing hepatic glycolysis reduces body weight and adiposity in mice (Wu et al. 2005). In Djungarian hamsters, photoperiod regulates the set‐point of body weight partially through changes in hypothalamic neuropeptide circuits controlling appetite and energy homeostasis (Morgan et al. 2003). The circuits are set early in life and induces persistent alterations in metabolic conditions, given that nutritional imbalances such as under‐ and overnutrition during critical ontogenic periods induce persistent modifications in the expression of the orexigenic/anorexigenic neuropeptides (Gali Ramamoorthy et al. 2015). In this study, postnatal photoperiod might program these hypothalamic neuropeptide circuits to determine the set‐point of body weight, and the glycolytic pathway was activated to maintain high body weight in 0–4 LD mice.

Metabolomic analysis further revealed that plasma levels of purine nucleosides (adenosine, inosine, and guanosine) and nucleotides (AMP, GMP, and IMP) were higher in 0–4 LD than in SDC groups, although levels of metabolites xanthine and uric acid were not altered. This suggests that purine nucleosides/nucleotides levels remain elevated without degrading them for excretion in 0–4 LD mice. It may be regulated by upregulated activity of IMP biosynthetic pathway and suppressed activity of purine nucleotide phosphorylase that metabolizes inosine to hypoxanthine and guanosine to guanine. Adenosine and guanosine have roles in protecting the nervous system both centrally and peripherally (Ribeiro et al. 2016) and the dysfunction of the purinergic system is linked to many symptoms of depression, bipolar disorder, and anxiety disorders, including dysfunctional sleep, cognitive impairment, and changes in appetite and energy levels (Ortiz et al. 2015). A link between the purinergic system and mood is confirmed in experimental animals, given that adenosine or inosine administration decreases depression‐like behaviors in mice (Kaster et al. 2004, 2013). Guanosine administration induces similar behavioral alterations with increased hippocampal neuronal differentiation (Bettio et al. 2016). These reports, along with our present data suggest that postnatal photoperiod alters mood‐related behaviors via alteration of the purinergic system. Indeed, we have preliminary data that the mice raised under LD until weaning exhibited lower depression‐like behaviors in adulthood with higher neurogenesis after weaning, compared to the mice raised under SD (Y. Takai, M. Kawai, M. Furuse, and S. Yasuo, unpubl. obs.). The central action of purinergic metabolites may also modify the structural circuit in the hypothalamus that controls appetite and energy balance, because perinatal undernutrition alters hypothalamic cell proliferation and axonal elongation of orexigenic/anorexigenic neurons (Gali Ramamoorthy et al. 2015).

Phosphocreatine serves as an energy reserve in the muscle. It is metabolized to creatine to produce ATP, followed by metabolism of creatine to creatinine (Wyss and Kaddurah‐Daouk 2000). Our results of low phosphocreatinine and high creatinine in 0–4 LD mice suggest that the metabolic pathway from phosphocreatine to creatinine was activated in these mice to maintain high body weight. Generally, creatinine levels are maintained at low levels by excretion to urine through kidney filtration (Wyss and Kaddurah‐Daouk 2000). Thus, the kidney function in the mice in 0–4 LD may be impaired than the mice in SDC. This concept is in line with higher levels of urea found in 0–4 LD mice than in SDC mice.

In conclusion, we demonstrated that postnatal exposure of mice to LD induces a persistent increase in body weight with various changes in the plasma metabolomics profile, compared to the mice maintained under SD. These data provide a key to understanding the link between photoperiod early in life and physiological phenotype in adulthood, and the correlation between season of birth and lifetime disease risk, including obesity, schizophrenia, bipolar disorder, major depression, and autism (Torrey et al. 1997; Wattie et al. 2008). Further studies are required to clarify the mechanisms underlying the metabolic programming by photoperiod.

## Conflict of Interest

None declared.

## Supporting information




**Table S1.** Plasma metabolites detected in the CE‐TOFMS.
