# Supplementary material for: Exposure of C57BL/6J mice to long photoperiod during early life stages increases body weight and alters plasma metabolomic profiles in adulthood
Source: Physiol Rep. 2016 Sep 20;4(18):e12974. doi: 10.14814/phy2.12974 (PMC5037922; doi:10.14814/phy2.12974)
Supplement: Supplementary file 1 — Table S1. Plasma metabolites detected in the CE‐TOFMS. [file PHY2-4-e12974-s001.pdf]

Supplemental Table 1. Plasma metabolites detected in the CE-TOFMS

| Compound name | Relative area | | | | | | Mean | | Comparative analysis | | |
|---|---|---|---|---|---|---|---|---|---|---|---|
| | SDC1 | SDC2 | SDC3 | 0-4LD1 | 0-4LD2 | 0-4LD3 | SDC | 0-4LD | Ratio* | *P*-value† | |
| Adenosine | 8.1E-05 | 7.1E-05 | 1.7E-04 | 9.5E-04 | 1.4E-03 | 3.1E-03 | 1.1E-04 | 1.8E-03 | 17 | 0.0611 | |
| Inosine | 1.7E-04 | 2.2E-04 | 1.3E-03 | 4.8E-03 | 2.5E-03 | 3.9E-03 | 5.5E-04 | 3.8E-03 | 6.8 | 0.0133 | # |
| IMP | 4.8E-05 | N.D. | 6.3E-05 | 4.4E-04 | 1.3E-04 | 5.2E-04 | 5.6E-05 | 3.7E-04 | 6.6 | 0.1375 | |
| Serotonin | 8.1E-04 | 5.9E-04 | 1.7E-03 | 5.9E-03 | 3.8E-03 | 7.0E-03 | 1.0E-03 | 5.6E-03 | 5.4 | 0.0099 | ## |
| 3-(4)Hydroxyphenyl)propionic acid | 3.1E-04 | 3.6E-04 | 2.6E-04 | 1.1E-03 | 1.4E-03 | 1.3E-03 | 3.1E-04 | 1.2E-03 | 4.0 | 0.0006 | ### |
| 1-Methylhistamine | 4.8E-05 | 4.5E-05 | 6.6E-05 | 1.7E-04 | 1.6E-04 | 1.9E-04 | 5.3E-05 | 1.7E-04 | 3.3 | 0.0002 | ### |
| Guanosine | 3.1E-05 | N.D. | 7.9E-05 | 1.8E-04 | 1.3E-04 | 1.7E-04 | 5.5E-05 | 1.6E-04 | 2.9 | 0.0342 | # |
| 4-Acetamidobutanoic acid | 3.4E-04 | 2.8E-04 | 3.5E-04 | 6.1E-04 | 5.9E-04 | 6.2E-04 | 3.2E-04 | 6.1E-04 | 1.9 | 0.0003 | ### |
| Phosphoenolpyruvic acid | 1.9E-04 | 1.3E-04 | 1.5E-04 | 3.3E-04 | 2.1E-04 | 3.2E-04 | 1.5E-04 | 2.9E-04 | 1.9 | 0.0352 | # |
| β-Ala | 3.7E-04 | 3.8E-04 | 3.9E-04 | 5.9E-04 | 6.0E-04 | 8.1E-04 | 3.8E-04 | 6.6E-04 | 1.7 | 0.0187 | # |
| Phosphorylcholine | 5.6E-04 | 7.1E-04 | 5.7E-04 | 1.1E-03 | 9.2E-04 | 1.2E-03 | 6.1E-04 | 1.1E-03 | 1.7 | 0.0080 | ## |
| Pyruvic acid | 5.3E-03 | 4.9E-03 | 4.3E-03 | 6.8E-03 | 8.3E-03 | 9.5E-03 | 4.8E-03 | 8.2E-03 | 1.7 | 0.0157 | # |
| Isovalerylalanine-1<br>N-Acetylleucine-1 | 1.8E-04 | 1.8E-04 | 5.5E-04 | 8.5E-04 | 4.2E-04 | 2.2E-04 | 3.0E-04 | 5.0E-04 | 1.6 | 0.4376 | |
| Glycerophosphocholine | 2.2E-03 | 1.5E-03 | 2.2E-03 | 3.1E-03 | 2.6E-03 | 3.9E-03 | 2.0E-03 | 3.2E-03 | 1.6 | 0.0505 | |
| *N*-Formylmethionine | 1.1E-04 | 8.2E-05 | 9.9E-05 | 1.3E-04 | 1.7E-04 | 1.6E-04 | 9.6E-05 | 1.5E-04 | 1.6 | 0.0174 | # |
| Anserine_divalent | 6.7E-04 | 5.5E-04 | 7.1E-04 | 1.1E-03 | 7.5E-04 | 1.2E-03 | 6.4E-04 | 1.0E-03 | 1.6 | 0.0631 | |
| Isovalerylalanine-2<br>N-Acetylleucine-2 | 2.2E-04 | 2.9E-04 | 1.9E-04 | 3.2E-04 | 3.6E-04 | 4.3E-04 | 2.3E-04 | 3.7E-04 | 1.6 | 0.0340 | # |
| *N*-Acetylaspartic acid | 1.6E-04 | 2.4E-04 | 3.8E-04 | 5.3E-04 | 2.8E-04 | 4.1E-04 | 2.6E-04 | 4.0E-04 | 1.6 | 0.1989 | |
| Ethanolamine phosphate | 1.9E-04 | 2.9E-04 | 3.0E-04 | 4.1E-04 | 3.9E-04 | 4.1E-04 | 2.6E-04 | 4.0E-04 | 1.6 | 0.0171 | # |
| Ribulose 5-phosphate | 1.2E-03 | 8.4E-04 | 1.7E-03 | 2.4E-03 | 1.5E-03 | 1.9E-03 | 1.3E-03 | 2.0E-03 | 1.6 | 0.1197 | |
| 1-Methyladenosine | 5.4E-05 | 5.1E-05 | 4.4E-05 | 9.4E-05 | 6.1E-05 | 7.2E-05 | 5.0E-05 | 7.6E-05 | 1.5 | 0.0601 | |
| Taurine | 1.9E-02 | 1.7E-02 | 1.7E-02 | 3.0E-02 | 2.0E-02 | 2.9E-02 | 1.8E-02 | 2.6E-02 | 1.5 | 0.0627 | |
| Glycerol 3-phosphate | 8.7E-04 | 8.7E-04 | 9.8E-04 | 1.3E-03 | 1.1E-03 | 1.6E-03 | 9.1E-04 | 1.3E-03 | 1.5 | 0.0333 | # |
| Homocarnosine | 1.9E-04 | 1.9E-04 | 2.0E-04 | 3.7E-04 | 2.0E-04 | 2.9E-04 | 2.0E-04 | 2.9E-04 | 1.5 | 0.1443 | |
| *p*-Hydroxybenzoic acid | 3.0E-04 | 3.6E-04 | 1.9E-04 | 4.0E-04 | 4.0E-04 | 4.2E-04 | 2.8E-04 | 4.1E-04 | 1.4 | 0.0772 | |
| Spermidine | 3.1E-05 | 4.7E-05 | 7.5E-05 | 8.9E-05 | 5.3E-05 | 7.7E-05 | 5.1E-05 | 7.3E-05 | 1.4 | 0.2601 | |
| *N*-Acetylasparagine | 7.9E-05 | 8.5E-05 | N.D. | 9.4E-05 | 1.4E-04 | 1.2E-04 | 8.2E-05 | 1.2E-04 | 1.4 | 0.1159 | |
| Guanidinosuccinic acid | 1.3E-04 | 1.2E-04 | 1.4E-04 | 2.0E-04 | 1.9E-04 | 1.7E-04 | 1.3E-04 | 1.9E-04 | 1.4 | 0.0031 | ## |
| Creatine | 7.0E-02 | 6.3E-02 | 6.2E-02 | 1.1E-01 | 6.6E-02 | 1.0E-01 | 6.5E-02 | 9.2E-02 | 1.4 | 0.1207 | |
| Carnosine | 2.4E-04 | 2.5E-04 | 2.6E-04 | 4.1E-04 | 2.5E-04 | 3.9E-04 | 2.5E-04 | 3.5E-04 | 1.4 | 0.1144 | |
| Thiamine phosphate | 6.8E-05 | 6.4E-05 | 6.9E-05 | 9.4E-05 | 7.9E-05 | 1.1E-04 | 6.7E-05 | 9.4E-05 | 1.4 | 0.0396 | # |
| Phenylpyruvic acid | 1.3E-04 | 1.6E-04 | N.D. | N.D. | 2.1E-04 | 2.0E-04 | 1.5E-04 | 2.0E-04 | 1.4 | 0.0636 | |
| Diethanolamine | 1.9E-04 | 2.3E-04 | 1.7E-04 | 2.6E-04 | 2.8E-04 | 2.7E-04 | 2.0E-04 | 2.7E-04 | 1.4 | 0.0169 | # |
| Glu | 1.4E-02 | 1.6E-02 | 1.2E-02 | 2.0E-02 | 1.6E-02 | 2.1E-02 | 1.4E-02 | 1.9E-02 | 1.4 | 0.0516 | |
| Ectoine | 5.5E-04 | 5.1E-04 | 3.3E-04 | 4.0E-04 | 6.8E-04 | 8.0E-04 | 4.6E-04 | 6.3E-04 | 1.4 | 0.2910 | |
| Galactosamine<br>Glucosamine | 1.0E-04 | 1.2E-04 | 9.7E-05 | 1.6E-04 | 1.3E-04 | N.D. | 1.1E-04 | 1.4E-04 | 1.4 | 0.0946 | |
| *N*-Acetyltryptophan | 1.4E-04 | 1.5E-04 | 1.2E-04 | 1.8E-04 | 1.8E-04 | 1.9E-04 | 1.4E-04 | 1.8E-04 | 1.3 | 0.0086 | ## |
| Homocitrulline | 4.8E-04 | 3.6E-04 | 3.8E-04 | 5.5E-04 | 5.9E-04 | 4.8E-04 | 4.1E-04 | 5.4E-04 | 1.3 | 0.0557 | |
| *N*-Acetylglutamine | 1.7E-04 | 1.5E-04 | 1.2E-04 | 1.7E-04 | 1.4E-04 | 2.7E-04 | 1.4E-04 | 1.9E-04 | 1.3 | 0.3203 | |
| *N*-Acetylglycine | 5.2E-04 | 4.8E-04 | 7.0E-04 | 9.8E-04 | 6.1E-04 | 6.3E-04 | 5.6E-04 | 7.4E-04 | 1.3 | 0.2763 | |
| XA0013 | 3.3E-03 | 3.4E-03 | 2.9E-03 | 3.8E-03 | 3.8E-03 | 4.8E-03 | 3.2E-03 | 4.1E-03 | 1.3 | 0.0724 | |
| Asp | 2.4E-03 | 5.0E-03 | 2.3E-03 | 4.4E-03 | 3.0E-03 | 5.0E-03 | 3.2E-03 | 4.1E-03 | 1.3 | 0.4388 | |
| Choline | 1.3E-02 | 1.4E-02 | 1.1E-02 | 1.7E-02 | 1.4E-02 | 1.7E-02 | 1.3E-02 | 1.6E-02 | 1.3 | 0.0581 | |

Supplemental Table 1 (Continued)

| Compound name | Relative area | | | | | | Mean | | Comparative analysis | | |
|---|---|---|---|---|---|---|---|---|---|---|---|
| | SDC1 | SDC2 | SDC3 | 0-4LD1 | 0-4LD2 | 0-4LD3 | SDC | 0-4LD | Ratio* | *P*-value† | |
| 3-Ureidopropionic acid | 1.4E-04 | 2.9E-04 | 1.9E-04 | 2.0E-04 | 3.3E-04 | 2.5E-04 | 2.1E-04 | 2.6E-04 | 1.3 | 0.4146 | |
| Isocitric acid | 2.1E-03 | 2.1E-03 | 2.3E-03 | 2.7E-03 | 2.6E-03 | 2.7E-03 | 2.2E-03 | 2.7E-03 | 1.2 | 0.0009 | ### |
| Ile | 5.6E-02 | 6.7E-02 | 5.0E-02 | 6.8E-02 | 6.7E-02 | 7.6E-02 | 5.7E-02 | 7.1E-02 | 1.2 | 0.0833 | |
| *S*-Sulfocysteine | 3.4E-04 | 6.1E-04 | 5.9E-04 | 5.9E-04 | 4.8E-04 | 8.2E-04 | 5.1E-04 | 6.3E-04 | 1.2 | 0.4305 | |
| Val | 9.9E-02 | 1.1E-01 | 8.4E-02 | 1.1E-01 | 1.2E-01 | 1.3E-01 | 9.8E-02 | 1.2E-01 | 1.2 | 0.1110 | |
| Leu | 9.8E-02 | 1.1E-01 | 8.4E-02 | 1.1E-01 | 1.2E-01 | 1.3E-01 | 9.8E-02 | 1.2E-01 | 1.2 | 0.0769 | |
| Trigonelline | 2.6E-03 | 2.4E-03 | 1.5E-03 | 1.8E-03 | 2.8E-03 | 3.3E-03 | 2.2E-03 | 2.6E-03 | 1.2 | 0.4396 | |
| Pipecolic acid | 2.2E-03 | 2.1E-03 | 2.1E-03 | 2.7E-03 | 2.3E-03 | 2.8E-03 | 2.1E-03 | 2.6E-03 | 1.2 | 0.0301 | # |
| 2-Oxoglutaric acid | 6.3E-03 | 7.5E-03 | 6.8E-03 | 7.0E-03 | 9.3E-03 | 8.5E-03 | 6.9E-03 | 8.3E-03 | 1.2 | 0.1369 | |
| Ethanolamine | 1.3E-03 | 1.4E-03 | 1.1E-03 | 1.5E-03 | 1.4E-03 | 1.7E-03 | 1.3E-03 | 1.5E-03 | 1.2 | 0.0847 | |
| Arg | 3.6E-02 | 4.5E-02 | 3.4E-02 | 3.6E-02 | 4.3E-02 | 5.8E-02 | 3.9E-02 | 4.6E-02 | 1.2 | 0.3610 | |
| 1-Methyl-4-imidazoleacetic acid | 2.1E-04 | 3.7E-04 | 2.3E-04 | 3.3E-04 | 2.3E-04 | 4.0E-04 | 2.7E-04 | 3.2E-04 | 1.2 | 0.5017 | |
| *O*-Acetylhomoserine<br>2-Aminoadipic acid | 1.2E-03 | 1.6E-03 | 1.4E-03 | 1.7E-03 | 1.5E-03 | 1.7E-03 | 1.4E-03 | 1.7E-03 | 1.2 | 0.0862 | |
| XA0012 | 2.8E-04 | 2.3E-04 | 2.4E-04 | 3.1E-04 | 3.1E-04 | 2.8E-04 | 2.5E-04 | 3.0E-04 | 1.2 | 0.0484 | # |
| Isethionic acid | 7.5E-04 | 5.5E-04 | 6.0E-04 | 8.9E-04 | 7.5E-04 | 6.1E-04 | 6.3E-04 | 7.5E-04 | 1.2 | 0.3130 | |
| Thiamine | 1.0E-04 | 9.7E-05 | 1.2E-04 | 1.3E-04 | 1.5E-04 | 1.1E-04 | 1.1E-04 | 1.3E-04 | 1.2 | 0.2281 | |
| $N^5$-Ethylglutamine | 2.1E-03 | 1.8E-03 | 1.5E-03 | 1.8E-03 | 2.2E-03 | 2.4E-03 | 1.8E-03 | 2.1E-03 | 1.2 | 0.2348 | |
| Terephthalic acid | 2.4E-04 | 2.3E-04 | 2.4E-04 | 3.1E-04 | 2.5E-04 | 2.8E-04 | 2.4E-04 | 2.8E-04 | 1.2 | 0.0557 | |
| 11-Aminoundecanoic acid | 8.8E-05 | 2.1E-04 | 1.3E-04 | 2.2E-04 | 1.7E-04 | 1.1E-04 | 1.4E-04 | 1.7E-04 | 1.2 | 0.6395 | |
| 3-Phosphoglyceric acid | 3.4E-04 | 1.8E-04 | 3.4E-04 | 4.5E-04 | 2.3E-04 | 3.3E-04 | 2.8E-04 | 3.3E-04 | 1.2 | 0.5916 | |
| Kynurenine | 4.3E-04 | 3.6E-04 | 3.7E-04 | 3.9E-04 | 5.2E-04 | 4.5E-04 | 3.9E-04 | 4.5E-04 | 1.2 | 0.2233 | |
| Trimethylamine *N*-oxide | 3.4E-03 | 3.0E-03 | 3.2E-03 | 3.2E-03 | 3.8E-03 | 4.2E-03 | 3.2E-03 | 3.7E-03 | 1.2 | 0.1486 | |
| *cis*-Aconitic acid | 2.1E-03 | 1.9E-03 | 2.0E-03 | 2.0E-03 | 2.4E-03 | 2.5E-03 | 2.0E-03 | 2.3E-03 | 1.2 | 0.1308 | |
| *N*-Acetylmethionine | 1.2E-04 | 1.4E-04 | 6.6E-05 | 1.0E-04 | 1.4E-04 | 1.3E-04 | 1.1E-04 | 1.2E-04 | 1.2 | 0.5121 | |
| *N*-Acetylgalactosamine<br>*N*-Acetylmannosamine<br>*N*-Acetylglucosamine | N.D. | 3.0E-04 | 3.6E-04 | 3.9E-04 | 3.5E-04 | 4.1E-04 | 3.3E-04 | 3.8E-04 | 1.2 | 0.1780 | |
| Urocanic acid | 1.8E-04 | 4.5E-04 | 2.1E-04 | 3.9E-04 | 2.0E-04 | 3.8E-04 | 2.8E-04 | 3.2E-04 | 1.2 | 0.6972 | |
| Nicotinamide | 4.3E-04 | 4.6E-04 | 6.5E-04 | 7.2E-04 | 4.7E-04 | 5.8E-04 | 5.1E-04 | 5.9E-04 | 1.1 | 0.4907 | |
| 2'-Deoxycytidine | 2.7E-04 | 2.8E-04 | 2.4E-04 | 2.9E-04 | 2.9E-04 | 3.2E-04 | 2.6E-04 | 3.0E-04 | 1.1 | 0.0651 | |
| 4-Guanidinobutyric acid | 7.0E-04 | 6.5E-04 | 3.3E-04 | 4.5E-04 | 7.8E-04 | 6.9E-04 | 5.6E-04 | 6.4E-04 | 1.1 | 0.6306 | |
| XC0001 | 6.0E-05 | N.D. | 8.7E-05 | 7.6E-05 | 8.4E-05 | 9.2E-05 | 7.3E-05 | 8.4E-05 | 1.1 | 0.4456 | |
| 5-Oxoproline | 1.0E-03 | 1.5E-03 | 1.0E-03 | 1.4E-03 | 9.5E-04 | 1.6E-03 | 1.2E-03 | 1.3E-03 | 1.1 | 0.5721 | |
| Homovanillic acid | 2.1E-04 | 2.5E-04 | 1.8E-04 | 2.4E-04 | 2.6E-04 | 2.3E-04 | 2.2E-04 | 2.5E-04 | 1.1 | 0.2688 | |
| Urea | 9.0E-01 | 9.3E-01 | 8.8E-01 | 9.5E-01 | 1.1E+00 | 1.1E+00 | 9.0E-01 | 1.0E+00 | 1.1 | 0.0427 | # |
| *N*-Acetylhistidine | 1.7E-04 | 1.8E-04 | 9.7E-05 | 1.3E-04 | 1.7E-04 | 2.0E-04 | 1.5E-04 | 1.6E-04 | 1.1 | 0.6175 | |
| Met | 1.0E-02 | 1.1E-02 | 8.5E-03 | 9.1E-03 | 1.2E-02 | 1.3E-02 | 9.9E-03 | 1.1E-02 | 1.1 | 0.4034 | |
| Carboxymethyllysine | 1.9E-04 | 1.2E-04 | 1.0E-04 | 1.5E-04 | 1.6E-04 | 1.4E-04 | 1.4E-04 | 1.5E-04 | 1.1 | 0.5731 | |
| XC0016 | 3.8E-04 | 3.3E-04 | 2.8E-04 | 3.2E-04 | 3.8E-04 | 4.0E-04 | 3.3E-04 | 3.7E-04 | 1.1 | 0.3669 | |
| Pantothenic acid | 2.5E-04 | 2.2E-04 | 2.2E-04 | 2.6E-04 | 2.7E-04 | 2.4E-04 | 2.3E-04 | 2.6E-04 | 1.1 | 0.1481 | |
| 3-Indoxylsulfuric acid | 4.5E-03 | 6.3E-03 | 4.6E-03 | 4.4E-03 | 5.2E-03 | 7.3E-03 | 5.1E-03 | 5.7E-03 | 1.1 | 0.6362 | |
| Pro | 3.2E-02 | 3.9E-02 | 2.2E-02 | 2.8E-02 | 3.2E-02 | 4.2E-02 | 3.1E-02 | 3.4E-02 | 1.1 | 0.6543 | |
| Lys | 6.2E-02 | 7.8E-02 | 5.6E-02 | 6.4E-02 | 6.4E-02 | 8.8E-02 | 6.5E-02 | 7.2E-02 | 1.1 | 0.5510 | |
| 2-Oxoisovaleric acid | 8.5E-04 | 9.8E-04 | 1.4E-03 | 1.2E-03 | 1.1E-03 | 1.2E-03 | 1.1E-03 | 1.2E-03 | 1.1 | 0.5752 | |
| 2-Hydroxyglutaric acid | 6.1E-04 | 7.1E-04 | 5.2E-04 | 4.8E-04 | 7.2E-04 | 7.9E-04 | 6.1E-04 | 6.7E-04 | 1.1 | 0.6330 | |
| 5-Hydroxylysine | 4.0E-04 | 5.2E-04 | 2.8E-04 | 2.8E-04 | 4.4E-04 | 6.0E-04 | 4.0E-04 | 4.4E-04 | 1.1 | 0.7632 | |

Supplemental Table 1 (Continued)

| Compound name | Relative area | | | | | | Mean | | Comparative analysis | |
|---|---|---|---|---|---|---|---|---|---|---|
| | SDC1 | SDC2 | SDC3 | 0-4LD1 | 0-4LD2 | 0-4LD3 | SDC | 0-4LD | Ratio* | *P*-value† |
| Hypotaurine | 1.1E-03 | 1.3E-03 | 1.3E-03 | 1.2E-03 | 1.4E-03 | 1.5E-03 | 1.2E-03 | 1.4E-03 | 1.1 | 0.3575 |
| Creatinine | 3.8E-03 | 3.6E-03 | 3.7E-03 | 3.9E-03 | 4.1E-03 | 4.1E-03 | 3.7E-03 | 4.0E-03 | 1.1 | 0.0281 # |
| Trp | 4.3E-02 | 4.1E-02 | 3.4E-02 | 4.1E-02 | 4.4E-02 | 4.3E-02 | 4.0E-02 | 4.3E-02 | 1.1 | 0.3349 |
| Citric acid | 4.1E-02 | 4.2E-02 | 4.8E-02 | 4.7E-02 | 4.6E-02 | 4.8E-02 | 4.4E-02 | 4.7E-02 | 1.1 | 0.2059 |
| Cytidine | 5.1E-04 | 5.3E-04 | 5.0E-04 | 6.5E-04 | 5.1E-04 | 4.9E-04 | 5.1E-04 | 5.5E-04 | 1.1 | 0.5050 |
| Thr | 3.6E-02 | 4.1E-02 | 3.0E-02 | 3.2E-02 | 3.5E-02 | 4.7E-02 | 3.6E-02 | 3.8E-02 | 1.1 | 0.6919 |
| Ornithine | 2.0E-02 | 2.7E-02 | 1.5E-02 | 1.7E-02 | 2.0E-02 | 2.8E-02 | 2.0E-02 | 2.2E-02 | 1.1 | 0.8035 |
| Tyr | 3.0E-02 | 3.3E-02 | 2.0E-02 | 2.3E-02 | 3.0E-02 | 3.4E-02 | 2.8E-02 | 2.9E-02 | 1.1 | 0.7879 |
| $N_{\omega}$-Methylarginine | 1.2E-04 | 1.2E-04 | 8.8E-05 | 9.9E-05 | 1.1E-04 | 1.4E-04 | 1.1E-04 | 1.2E-04 | 1.1 | 0.7370 |
| Putrescine | 1.6E-04 | 1.6E-04 | 1.1E-04 | 1.4E-04 | 1.6E-04 | 1.4E-04 | 1.4E-04 | 1.5E-04 | 1.1 | 0.7015 |
| XC0040 | 1.8E-04 | 1.9E-04 | 1.2E-04 | N.D. | 1.8E-04 | 1.6E-04 | 1.6E-04 | 1.7E-04 | 1.0 | 0.8246 |
| γ-Glu-Val-Gly | 1.2E-04 | 7.3E-05 | 9.2E-05 | 6.8E-05 | 1.3E-04 | 1.1E-04 | 9.6E-05 | 1.0E-04 | 1.0 | 0.8694 |
| XA0035 | 2.8E-04 | 2.6E-04 | 3.3E-04 | 3.2E-04 | 3.0E-04 | 2.9E-04 | 2.9E-04 | 3.0E-04 | 1.0 | 0.6472 |
| Cysteine glutathione disulfide | 5.1E-03 | 5.6E-03 | 6.0E-03 | 5.3E-03 | 6.9E-03 | 5.0E-03 | 5.6E-03 | 5.7E-03 | 1.0 | 0.7702 |
| Butyrylcarnitine | 1.8E-03 | 1.8E-03 | 1.2E-03 | 1.2E-03 | 2.0E-03 | 1.8E-03 | 1.6E-03 | 1.7E-03 | 1.0 | 0.8613 |
| Ophthalmic acid | 2.5E-04 | 2.3E-04 | 1.9E-04 | 1.6E-04 | 3.6E-04 | 1.7E-04 | 2.2E-04 | 2.3E-04 | 1.0 | 0.9216 |
| XA0004 | 6.1E-05 | 7.8E-05 | 5.6E-05 | N.D. | 6.1E-05 | 7.3E-05 | 6.5E-05 | 6.7E-05 | 1.0 | 0.8853 |
| Phe | 3.3E-02 | 4.0E-02 | 2.7E-02 | 3.0E-02 | 3.5E-02 | 3.7E-02 | 3.3E-02 | 3.4E-02 | 1.0 | 0.8782 |
| Uridine | 1.2E-03 | 1.4E-03 | 1.5E-03 | 1.5E-03 | 1.3E-03 | 1.3E-03 | 1.4E-03 | 1.4E-03 | 1.0 | 0.7492 |
| Asn | 6.7E-03 | 9.6E-03 | 5.8E-03 | 7.2E-03 | 6.6E-03 | 8.9E-03 | 7.4E-03 | 7.6E-03 | 1.0 | 0.9073 |
| Cystathionine | 2.5E-04 | 2.2E-04 | 2.2E-04 | 2.1E-04 | 2.3E-04 | 2.7E-04 | 2.3E-04 | 2.4E-04 | 1.0 | 0.8164 |
| Gly | 4.7E-02 | 5.1E-02 | 4.5E-02 | 5.0E-02 | 4.1E-02 | 5.5E-02 | 4.8E-02 | 4.9E-02 | 1.0 | 0.8410 |
| *S*-Adenosylmethionine | 3.8E-05 | 2.6E-05 | 5.3E-05 | 4.4E-05 | 3.6E-05 | 3.9E-05 | 3.9E-05 | 4.0E-05 | 1.0 | 0.9297 |
| Glucuronic acid<br>Galacturonic acid | 5.1E-04 | 4.2E-04 | 5.0E-04 | 4.9E-04 | 4.3E-04 | 5.2E-04 | 4.7E-04 | 4.8E-04 | 1.0 | 0.8426 |
| SDMA | 1.5E-04 | 1.8E-04 | 1.5E-04 | 1.8E-04 | 1.6E-04 | 1.5E-04 | 1.6E-04 | 1.6E-04 | 1.0 | 0.8780 |
| 5-Methoxyindoleacetic acid | 2.2E-04 | 2.6E-04 | 2.2E-04 | 1.4E-04 | 2.9E-04 | 2.9E-04 | 2.4E-04 | 2.4E-04 | 1.0 | 0.9541 |
| Gluconolactone | 1.4E-03 | 1.1E-03 | 1.2E-03 | 1.3E-03 | 1.4E-03 | 1.2E-03 | 1.2E-03 | 1.3E-03 | 1.0 | 0.9689 |
| Cystine | 1.5E-03 | 2.2E-03 | 2.4E-03 | 2.1E-03 | 2.4E-03 | 1.7E-03 | 2.1E-03 | 2.1E-03 | 1.0 | 0.9992 |
| Ala | 5.6E-02 | 7.0E-02 | 4.3E-02 | 4.8E-02 | 5.5E-02 | 6.6E-02 | 5.6E-02 | 5.6E-02 | 1.0 | 0.9826 |
| Imidazolelactic acid | 1.2E-04 | 9.4E-05 | 9.7E-05 | 1.0E-04 | 1.0E-04 | 1.1E-04 | 1.0E-04 | 1.0E-04 | 1.0 | 0.9603 |
| Glutathione (GSSG)_divalent | 5.9E-03 | 6.1E-03 | 5.9E-03 | 5.2E-03 | 6.8E-03 | 5.8E-03 | 6.0E-03 | 5.9E-03 | 1.0 | 0.9399 |
| Lauric acid | 2.8E-03 | 2.5E-03 | 2.5E-03 | 2.6E-03 | 2.4E-03 | 2.6E-03 | 2.6E-03 | 2.5E-03 | 1.0 | 0.8078 |
| 2-Hydroxybutyric acid | 1.4E-03 | 1.8E-03 | 2.1E-03 | 1.8E-03 | 1.8E-03 | 1.6E-03 | 1.8E-03 | 1.7E-03 | 1.0 | 0.9296 |
| *o*-Hydroxybenzoic acid | 4.5E-04 | 6.0E-04 | 3.3E-04 | 2.1E-04 | 5.5E-04 | 6.1E-04 | 4.6E-04 | 4.5E-04 | 1.0 | 0.9722 |
| Lactic acid | 4.0E-01 | 4.1E-01 | 3.4E-01 | 3.3E-01 | 4.0E-01 | 4.0E-01 | 3.8E-01 | 3.8E-01 | 1.0 | 0.8899 |
| Argininosuccinic acid | 1.1E-04 | 1.5E-04 | 9.1E-05 | 8.6E-05 | 9.6E-05 | 1.7E-04 | 1.2E-04 | 1.2E-04 | 1.0 | 0.9534 |
| *N*-Acetylalanine | 1.6E-04 | 1.5E-04 | 1.4E-04 | 1.6E-04 | 1.7E-04 | 1.1E-04 | 1.5E-04 | 1.5E-04 | 1.0 | 0.8993 |
| Gln | 1.2E-01 | 1.2E-01 | 1.1E-01 | 1.3E-01 | 9.8E-02 | 1.1E-01 | 1.2E-01 | 1.1E-01 | 1.0 | 0.8321 |
| Phenaceturic acid | 4.6E-04 | 3.7E-04 | 3.3E-04 | 4.1E-04 | 2.2E-04 | 5.1E-04 | 3.9E-04 | 3.8E-04 | 1.0 | 0.9408 |
| Stachydrine | 1.0E-02 | 7.9E-03 | 6.7E-03 | 6.6E-03 | 8.9E-03 | 8.6E-03 | 8.2E-03 | 8.0E-03 | 1.0 | 0.8538 |
| $N^6$-Acetyllysine | 3.5E-04 | 3.6E-04 | 3.4E-04 | 3.2E-04 | 3.9E-04 | 3.2E-04 | 3.5E-04 | 3.4E-04 | 1.0 | 0.7033 |
| 1-Methylhistidine<br>3-Methylhistidine | 4.1E-03 | 3.8E-03 | 4.0E-03 | 4.1E-03 | 3.4E-03 | 4.2E-03 | 4.0E-03 | 3.9E-03 | 1.0 | 0.6744 |
| $N^6$-Methyllysine | 3.2E-03 | 3.2E-03 | 2.9E-03 | 2.7E-03 | 3.1E-03 | 3.3E-03 | 3.1E-03 | 3.0E-03 | 1.0 | 0.6727 |

Supplemental Table 1 (Continued)

| Compound name | Relative area | | | | | | Mean | | Comparative analysis | | |
|---|---|---|---|---|---|---|---|---|---|---|---|
| | SDC1 | SDC2 | SDC3 | 0-4LD1 | 0-4LD2 | 0-4LD3 | SDC | 0-4LD | Ratio* | *P*-value† | |
| 4-Methyl-2-oxovaleric acid<br>3-Methyl-2-oxovaleric acid | 3.6E-03 | 3.3E-03 | 5.1E-03 | 4.4E-03 | 3.4E-03 | 3.7E-03 | 4.0E-03 | 3.8E-03 | 1.0 | 0.7927 | |
| Myristoleic acid | 6.7E-05 | N.D. | 7.9E-05 | 9.9E-05 | N.D. | 4.0E-05 | 7.3E-05 | 6.9E-05 | 1.0 | 0.9234 | |
| 2'-Deoxyuridine | 3.1E-04 | 2.4E-04 | 3.2E-04 | 2.6E-04 | 3.0E-04 | 2.6E-04 | 2.9E-04 | 2.7E-04 | 0.9 | 0.5866 | |
| Gluconic acid | 2.8E-03 | 2.2E-03 | 2.6E-03 | 2.3E-03 | 2.4E-03 | 2.5E-03 | 2.5E-03 | 2.4E-03 | 0.9 | 0.4981 | |
| Uric acid | 4.8E-03 | 3.5E-03 | 4.0E-03 | 3.8E-03 | 3.4E-03 | 4.4E-03 | 4.1E-03 | 3.9E-03 | 0.9 | 0.6418 | |
| *N*,*N*-Dimethylglycine | 3.7E-03 | 3.3E-03 | 3.1E-03 | 3.1E-03 | 3.0E-03 | 3.4E-03 | 3.4E-03 | 3.2E-03 | 0.9 | 0.4084 | |
| Glycolic acid | 3.6E-03 | 2.8E-03 | 4.0E-03 | 3.6E-03 | 3.1E-03 | 3.1E-03 | 3.5E-03 | 3.3E-03 | 0.9 | 0.6181 | |
| Ser | 2.2E-02 | 2.4E-02 | 2.0E-02 | 2.0E-02 | 1.8E-02 | 2.4E-02 | 2.2E-02 | 2.1E-02 | 0.9 | 0.5203 | |
| Citrulline | 1.9E-02 | 2.5E-02 | 1.9E-02 | 1.8E-02 | 1.6E-02 | 2.2E-02 | 2.1E-02 | 1.9E-02 | 0.9 | 0.5274 | |
| Thymidine | 5.0E-04 | 4.7E-04 | 4.5E-04 | 3.9E-04 | 4.3E-04 | 4.7E-04 | 4.7E-04 | 4.3E-04 | 0.9 | 0.1861 | |
| Hydroxyproline | 6.4E-03 | 7.2E-03 | 5.2E-03 | 4.8E-03 | 5.4E-03 | 6.7E-03 | 6.2E-03 | 5.6E-03 | 0.9 | 0.4879 | |
| XC0120 | 3.5E-05 | 6.1E-05 | 3.7E-05 | 3.8E-05 | 4.5E-05 | 3.6E-05 | 4.4E-05 | 4.0E-05 | 0.9 | 0.6443 | |
| Threonic acid | 3.6E-03 | 2.9E-03 | 4.3E-03 | 3.7E-03 | 3.0E-03 | 3.0E-03 | 3.6E-03 | 3.2E-03 | 0.9 | 0.4971 | |
| Decanoic acid | 3.1E-04 | N.D. | 2.6E-04 | 2.5E-04 | N.D. | 2.6E-04 | 2.8E-04 | 2.5E-04 | 0.9 | 0.3742 | |
| Succinic acid | 5.4E-03 | 7.2E-03 | 5.7E-03 | 5.0E-03 | 5.6E-03 | 5.7E-03 | 6.1E-03 | 5.4E-03 | 0.9 | 0.3355 | |
| 2-Hydroxyvaleric acid | 3.5E-03 | 3.0E-03 | 4.0E-03 | 3.2E-03 | 2.8E-03 | 3.5E-03 | 3.5E-03 | 3.1E-03 | 0.9 | 0.3372 | |
| 2-Aminoisobutyric acid<br>2-Aminobutyric acid | 1.6E-03 | 1.9E-03 | 1.5E-03 | 1.4E-03 | 1.4E-03 | 1.6E-03 | 1.7E-03 | 1.5E-03 | 0.9 | 0.2212 | |
| His | 2.4E-02 | 2.6E-02 | 2.0E-02 | 2.1E-02 | 2.0E-02 | 2.2E-02 | 2.3E-02 | 2.1E-02 | 0.9 | 0.2259 | |
| 2-Hydroxyisobutyric acid | 1.3E-03 | 1.3E-03 | 1.7E-03 | 1.5E-03 | 9.6E-04 | 1.4E-03 | 1.4E-03 | 1.3E-03 | 0.9 | 0.4405 | |
| 3-Hydroxybutyric acid | 1.3E-02 | 1.6E-02 | 3.6E-02 | 2.8E-02 | 1.5E-02 | 1.4E-02 | 2.2E-02 | 1.9E-02 | 0.9 | 0.7876 | |
| Fumaric acid | 1.2E-03 | 1.5E-03 | 1.1E-03 | 1.0E-03 | 9.4E-04 | 1.4E-03 | 1.3E-03 | 1.1E-03 | 0.9 | 0.4887 | |
| 7,8-Dihydrobiopterin | N.D. | 4.4E-05 | 5.6E-05 | 4.1E-05 | 4.7E-05 | 4.3E-05 | 5.0E-05 | 4.4E-05 | 0.9 | 0.3115 | |
| Carnitine | 2.8E-02 | 2.4E-02 | 2.2E-02 | 2.2E-02 | 1.9E-02 | 2.3E-02 | 2.5E-02 | 2.2E-02 | 0.9 | 0.1969 | |
| Malic acid | 1.6E-02 | 2.4E-02 | 1.7E-02 | 1.5E-02 | 1.4E-02 | 2.0E-02 | 1.9E-02 | 1.6E-02 | 0.9 | 0.4873 | |
| 2-Hydroxy-4-methylvaleric acid | 9.2E-04 | 8.0E-04 | 7.9E-04 | 7.5E-04 | 6.6E-04 | 7.6E-04 | 8.4E-04 | 7.2E-04 | 0.9 | 0.1060 | |
| Guanidoacetic acid | 8.2E-04 | 1.1E-03 | 9.2E-04 | 7.4E-04 | 8.3E-04 | 8.7E-04 | 9.4E-04 | 8.1E-04 | 0.9 | 0.2137 | |
| Isovalerylcarnitine | 3.0E-04 | 3.7E-04 | 2.5E-04 | 2.3E-04 | 2.5E-04 | 3.1E-04 | 3.1E-04 | 2.6E-04 | 0.8 | 0.3509 | |
| *N*-Acetylmuramic acid | 9.5E-05 | 9.6E-05 | N.D. | 5.2E-05 | 7.7E-05 | 1.1E-04 | 9.5E-05 | 8.0E-05 | 0.8 | 0.5461 | |
| *O*-Acetylcarnitine | 2.6E-02 | 2.3E-02 | 3.0E-02 | 2.7E-02 | 2.0E-02 | 1.9E-02 | 2.6E-02 | 2.2E-02 | 0.8 | 0.2280 | |
| GABA | 2.3E-04 | 4.3E-04 | 1.6E-04 | 2.3E-04 | 2.3E-04 | 2.2E-04 | 2.7E-04 | 2.2E-04 | 0.8 | 0.5739 | |
| Glucose 6-phosphate | 3.0E-04 | 3.3E-04 | 3.2E-04 | 3.0E-04 | 2.8E-04 | 1.9E-04 | 3.2E-04 | 2.6E-04 | 0.8 | 0.1626 | |
| Mucic acid | 5.2E-04 | 4.6E-04 | 4.7E-04 | 4.1E-04 | 3.8E-04 | 3.7E-04 | 4.8E-04 | 3.8E-04 | 0.8 | 0.0146 | # |
| Glyceric acid | 7.5E-04 | 6.2E-04 | 5.9E-04 | 4.5E-04 | 5.2E-04 | 5.4E-04 | 6.6E-04 | 5.1E-04 | 0.8 | 0.0556 | |
| γ-Butyrobetaine | 2.1E-03 | 2.0E-03 | 1.7E-03 | 1.5E-03 | 1.4E-03 | 1.5E-03 | 1.9E-03 | 1.5E-03 | 0.8 | 0.0261 | # |
| Pyridoxal | 1.2E-04 | 1.8E-04 | 1.3E-04 | 8.9E-05 | 1.2E-04 | 1.1E-04 | 1.4E-04 | 1.1E-04 | 0.7 | 0.1440 | |
| Sarcosine | 7.3E-04 | 7.5E-04 | N.D. | 3.8E-04 | 5.1E-04 | 7.7E-04 | 7.4E-04 | 5.5E-04 | 0.7 | 0.2868 | |
| XC0061 | 1.8E-03 | 2.0E-03 | 1.2E-03 | 1.1E-03 | 1.3E-03 | 1.2E-03 | 1.7E-03 | 1.2E-03 | 0.7 | 0.1317 | |
| Betaine | 5.1E-02 | 4.1E-02 | 3.5E-02 | 2.7E-02 | 2.8E-02 | 3.0E-02 | 4.2E-02 | 2.8E-02 | 0.7 | 0.0386 | # |
| *S*-Methylmethionine | 1.5E-04 | 1.2E-04 | 1.3E-04 | 7.9E-05 | 9.6E-05 | 9.4E-05 | 1.4E-04 | 9.0E-05 | 0.7 | 0.0129 | # |
| Glycerol | 3.3E-02 | 3.2E-02 | 4.8E-02 | 2.5E-02 | 2.2E-02 | 2.3E-02 | 3.7E-02 | 2.4E-02 | 0.6 | 0.0558 | |
| *N*-Acetyl-β-alanine | N.D. | 6.3E-05 | 7.5E-05 | 3.4E-05 | 2.6E-05 | N.D. | 6.9E-05 | 3.0E-05 | 0.4 | 0.0352 | # |
| *N*-Acetylphenylalanine | 2.5E-04 | 3.3E-04 | 2.0E-04 | 1.1E-04 | 1.1E-04 | 1.3E-04 | 2.6E-04 | 1.1E-04 | 0.4 | 0.0184 | # |
| Phosphocreatine | 5.6E-04 | 4.3E-04 | 4.4E-04 | 8.0E-05 | 2.6E-04 | 1.2E-04 | 4.8E-04 | 1.5E-04 | 0.3 | 0.0080 | ## |
| 3-Phenylpropionic acid | 7.7E-04 | 1.0E-03 | 7.6E-04 | 2.1E-04 | 1.9E-04 | 2.6E-04 | 8.5E-04 | 2.2E-04 | 0.3 | 0.0016 | ## |
| Cholic acid | 5.9E-04 | 5.1E-03 | 5.0E-04 | 4.7E-04 | 2.6E-04 | 5.8E-04 | 2.1E-03 | 4.4E-04 | 0.2 | 0.3461 | |

Supplemental Table 1 (Continued)

| Compound name | Relative area | | | | | | Mean | | Comparative analysis | |
|---|---|---|---|---|---|---|---|---|---|---|
| | SDC1 | SDC2 | SDC3 | 0-4LD1 | 0-4LD2 | 0-4LD3 | SDC | 0-4LD | Ratio[a] | *P*-value[†] |
| Taurocholic acid | 3.9E-04 | 3.5E-03 | 2.1E-04 | 2.0E-04 | 2.3E-04 | 1.8E-04 | 1.4E-03 | 2.0E-04 | 0.15 | 0.3400 |
| Hippuric acid | 2.0E-03 | 1.6E-03 | 1.5E-03 | 1.0E-04 | 1.3E-04 | 1.7E-04 | 1.7E-03 | 1.4E-04 | 0.08 | 0.0005 ### |
| 1-Methylnicotinamide | N.D. | N.D. | N.D. | N.D. | 7.7E-05 | 8.5E-05 | N.D. | 8.1E-05 | - | - |
| 5-Aminovaleric acid | N.D. | N.D. | N.D. | N.D. | N.D. | 1.1E-03 | N.D. | 1.1E-03 | - | - |
| 6-Aminohexanoic acid | N.D. | N.D. | N.D. | N.D. | 2.5E-04 | 2.8E-04 | N.D. | 2.6E-04 | - | - |
| ADP | N.D. | N.D. | N.D. | 3.3E-04 | 3.1E-04 | 5.7E-04 | N.D. | 4.0E-04 | - | - |
| ATP | N.D. | N.D. | N.D. | N.D. | N.D. | 7.1E-05 | N.D. | 7.1E-05 | - | - |
| Adipic acid | N.D. | N.D. | N.D. | N.D. | N.D. | 1.0E-04 | N.D. | 1.0E-04 | - | - |
| Cyclohexylamine | N.D. | N.D. | N.D. | N.D. | 5.7E-05 | N.D. | N.D. | 5.7E-05 | - | - |
| Cytosine | N.D. | N.D. | N.D. | N.D. | N.D. | 2.2E-04 | N.D. | 2.2E-04 | - | - |
| Dihydroxyacetone phosphate | N.D. | N.D. | N.D. | 5.9E-04 | 1.9E-04 | 4.3E-04 | N.D. | 4.0E-04 | - | - |
| *N*-Ethylmaleimide_+$H_2O$ | N.D. | N.D. | N.D. | 1.9E-04 | N.D. | N.D. | N.D. | 1.9E-04 | - | - |
| γ-Glu-2-aminobutyric acid | N.D. | N.D. | N.D. | N.D. | N.D. | 6.7E-05 | N.D. | 6.7E-05 | - | - |
| 2,6-Diaminopimelic acid | N.D. | 1.3E-04 | N.D. | N.D. | N.D. | N.D. | 1.3E-04 | N.D. | - | - |
| 8-Hydroxyoctanoic acid<br>2-Hydroxyoctanoic acid | 5.4E-05 | N.D. | N.D. | N.D. | N.D. | N.D. | 5.4E-05 | N.D. | - | - |
| Acetoacetic acid | N.D. | N.D. | 2.9E-04 | N.D. | N.D. | N.D. | 2.9E-04 | N.D. | - | - |
| Glutaric acid | N.D. | 3.5E-04 | N.D. | N.D. | N.D. | N.D. | 3.5E-04 | N.D. | - | - |
| Isobutyric acid<br>Butyric acid | N.D. | 2.9E-03 | N.D. | N.D. | N.D. | N.D. | 2.9E-03 | N.D. | - | - |
| Piperidine | N.D. | N.D. | 3.3E-05 | N.D. | N.D. | N.D. | 3.3E-05 | N.D. | - | - |
| Propionic acid | N.D. | 1.4E-03 | N.D. | N.D. | N.D. | N.D. | 1.4E-03 | N.D. | - | - |
| XC0065 | 5.8E-05 | N.D. | N.D. | N.D. | N.D. | N.D. | 5.8E-05 | N.D. | - | - |
| AMP | N.D. | N.D. | 2.4E-04 | 4.3E-03 | 2.3E-03 | 4.9E-03 | 2.4E-04 | 3.8E-03 | 16 | - |
| GMP | N.D. | N.D. | 1.4E-04 | 8.1E-04 | 5.1E-04 | 9.1E-04 | 1.4E-04 | 7.4E-04 | 5.3 | - |
| UMP | N.D. | N.D. | 8.8E-05 | 3.3E-04 | 2.0E-04 | 5.4E-04 | 8.8E-05 | 3.5E-04 | 4.0 | - |
| XC0126 | N.D. | 3.6E-05 | N.D. | 6.5E-05 | 7.7E-05 | 5.3E-05 | 3.6E-05 | 6.5E-05 | 1.8 | - |
| XA0017 | N.D. | N.D. | 5.6E-05 | 1.1E-04 | 8.8E-05 | N.D. | 5.6E-05 | 1.0E-04 | 1.8 | - |
| Hypoxanthine | N.D. | N.D. | 5.0E-04 | 1.2E-03 | 6.5E-04 | 5.5E-04 | 5.0E-04 | 8.0E-04 | 1.6 | - |
| *p*-Coumaric acid | N.D. | 8.0E-05 | N.D. | 1.2E-04 | 1.6E-04 | 9.2E-05 | 8.0E-05 | 1.2E-04 | 1.5 | - |
| Glucaric acid | 6.6E-05 | N.D. | N.D. | 6.4E-05 | N.D. | 1.1E-04 | 6.6E-05 | 8.9E-05 | 1.4 | - |
| Xanthine | N.D. | N.D. | 4.9E-04 | 9.0E-04 | 5.5E-04 | 4.2E-04 | 4.9E-04 | 6.2E-04 | 1.3 | - |
| 2,5-Dihydroxybenzoic acid | N.D. | 6.0E-05 | N.D. | N.D. | 7.2E-05 | N.D. | 6.0E-05 | 7.2E-05 | 1.2 | - |
| Fructose 6-phosphate | N.D. | 6.8E-05 | N.D. | N.D. | 7.3E-05 | 8.4E-05 | 6.8E-05 | 7.9E-05 | 1.2 | - |
| 3-Hydroxykynurenine | 5.9E-05 | N.D. | N.D. | 5.7E-05 | 7.5E-05 | N.D. | 5.9E-05 | 6.6E-05 | 1.1 | - |
| Triethanolamine | N.D. | 8.8E-05 | 7.5E-05 | 8.3E-05 | N.D. | N.D. | 8.2E-05 | 8.3E-05 | 1.0 | - |
| XA0027 | N.D. | 3.4E-04 | N.D. | 3.3E-04 | 3.2E-04 | N.D. | 3.4E-04 | 3.3E-04 | 1.0 | - |
| XA0002 | 5.0E-04 | N.D. | N.D. | N.D. | N.D. | 4.2E-04 | 5.0E-04 | 4.2E-04 | 0.8 | - |
| Pelargonic acid | 4.3E-04 | N.D. | N.D. | 3.8E-04 | 3.7E-04 | 3.3E-04 | 4.3E-04 | 3.6E-04 | 0.8 | - |
| $N^8$-Acetylspermidine | N.D. | 5.4E-05 | 3.8E-05 | 3.7E-05 | N.D. | N.D. | 4.6E-05 | 3.7E-05 | 0.8 | - |
| XA0036<br>Ascorbate 2-sulfate | 8.4E-05 | N.D. | N.D. | 6.7E-05 | N.D. | 4.6E-05 | 8.4E-05 | 5.6E-05 | 0.7 | - |
| Indole-3-acetic acid | 9.5E-05 | 9.8E-05 | N.D. | 6.2E-05 | N.D. | N.D. | 9.7E-05 | 6.2E-05 | 0.6 | - |

[*]Ratio of 0-4LD to SDC

[†]t-test, #*p* < 0.05, ##*p*<0.01, ###*p* < 0.001
